# Inducing Task-Relevant Responses to Speech in the Sleeping Brain

**DOI:** 10.1016/j.cub.2014.08.016

**Published:** 2014-09-22

**Authors:** Sid Kouider, Thomas Andrillon, Leonardo S. Barbosa, Louise Goupil, Tristan A. Bekinschtein

**Affiliations:** 1 Laboratoire de Sciences Cognitives et Psycholinguistique, CNRS/EHESS/DEC-ENS, 29 Rue d’Ulm, 75005 Paris, France; 2 Ecole Doctorale Cerveau-Cognition-Comportement, Université Pierre et Marie Curie, 9 Quai Saint Bernard, 75005 Paris, France; 3 Cognition and Brain Sciences Unit, Medical Research Council, 15 Chaucer Road, Cambridge CB2 7EF, UK; 4 Department of Psychology, University of Cambridge, Downing Street, Cambridge CB2 3EB, UK

## Abstract

Falling asleep leads to a loss of sensory awareness and to the inability to interact with the environment [1]. While this was traditionally thought as a consequence of the brain shutting down to external inputs, it is now acknowledged that incoming stimuli can still be processed, at least to some extent, during sleep [2]. For instance, sleeping participants can create novel sensory associations between tones and odors [3] or reactivate existing semantic associations, as evidenced by event-related potentials [4, 5, 6, 7]. Yet, the extent to which the brain continues to process external stimuli remains largely unknown. In particular, it remains unclear whether sensory information can be processed in a flexible and task-dependent manner by the sleeping brain, all the way up to the preparation of relevant actions. Here, using semantic categorization and lexical decision tasks, we studied task-relevant responses triggered by spoken stimuli in the sleeping brain. Awake participants classified words as either animals or objects (experiment 1) or as either words or pseudowords (experiment 2) by pressing a button with their right or left hand, while transitioning toward sleep. The lateralized readiness potential (LRP), an electrophysiological index of response preparation, revealed that task-specific preparatory responses are preserved during sleep. These findings demonstrate that despite the absence of awareness and behavioral responsiveness, sleepers can still extract task-relevant information from external stimuli and covertly prepare for appropriate motor responses.

## Highlights

• Subjects classifying spoken words continue performing the task after falling asleep
• Movement-related brain activity in the absence of overt behavior is demonstrated
• The sleeping brain can process spoken words in a task-dependent manner
• Response preparation is slower in sleep than in wakefulness

## Teaser

Kouider et al. show that subjects doing semantic categorization of spoken words continue performing this task after falling asleep. Analyses of neural activity in sleep shows a motor preparation bias for the correct response hand in the absence of overt behavior. Thus, the sleeping brain can process external stimuli in a task-dependent manner.

## Results

We studied whether the categorization of spoken words can still trigger task-relevant motor plans during early sleep stages. One main difficulty in addressing this issue consists in instructing a new task to sleeping subjects, arguably because prefrontal regions dealing with executive functions are then particularly suppressed in comparison to other cortical regions [8, 9]. One potential solution is to rely on the induction approach commonly used by studies on implicit perception in awake participants. This research reveals that the processing stream involved in making a semantic classification can, through explicit practice, be automatized and bypass prefrontal regions. Under those conditions, the categorization of visual words and numbers can lead to the covert activation of motor cortex even when those stimuli are masked and presented below the threshold of consciousness [10, 11]. In the current study, we extend this task induction strategy to track the ability of sleepers in extracting task-relevant information from speech and preparing for the appropriate motor plan.

### LRPs Reveal Semantic Classification and Response Preparation before and after Falling Asleep

**Figure 1:**
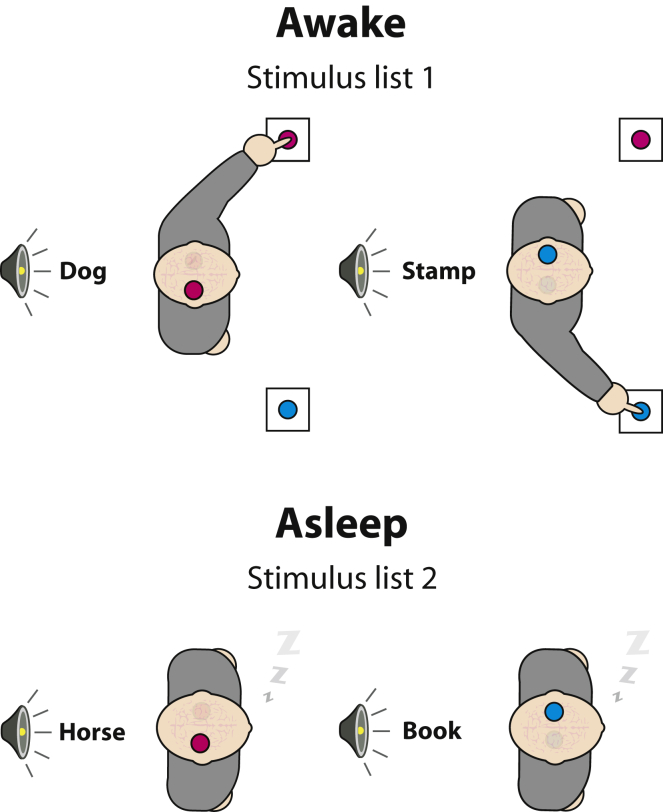
Schematic Description of the Induction Procedure (A) Participants (n = 18 in each experiment) first made overt manual responses either to animal versus object names presented every 6 to 9 s (semantic decision task; experiment 1), or to words versus pseudowords (lexical decision task; experiment 2) while wearing an EEG cap. (B) After participants either fell asleep (experiment 1) or entered the N2 stage (experiment 2), as assessed both by the absence of behavioral responses and by electrophysiological markers of sleep, a second list of stimuli was presented, and EEG indices of response preparation were used to evaluate covert classification. See also Figures S1 and S4.

We recorded the electroencephalogram (EEG) of human participants while they were awake and instructed them to classify spoken words as animals or objects (Figure 1). This procedure allowed us to compute lateralized readiness potentials (LRPs)—a neural marker of response selection and preparation [12]—by mapping each specific semantic category to a specific motor plan (e.g., animals with the right hand and objects with the left hand, counterbalanced across participants). This design allows for the assessment of lateralized response preparation toward the side associated with the appropriate semantic category. Thus, it allows for testing of whether sensory signals are processed beyond semantic levels by probing how the meaning extracted from external words can lead to the covert selection and preparation of context-dependent actions. Testing conditions encouraged the transition toward sleep while remaining engaged with the same task set: subjects received explicit allowance to fall asleep and were sitting in a dark room, eyes closed, in a reclining chair, listening to several repetitions of the same list of stimuli with a long intertrial interval of 6–9 s. Crucially, participants received an entirely new list of words (n = 48) during sleep to ensure that their responses were based on the extraction of word meaning rather than a mere reactivation of stimulus-response associations established during the wake stage.

Sleep onset was assessed online both behaviorally, by ensuring the absence of overt responses for at least 2 min of stimulation, and electrophysiologically, through sleep markers (i.e., disappearance of low-amplitude alpha/beta rhythms and development of high-amplitude delta/theta rhythms [see Figure S1 available online], presence of slow eye movements and other sleep graphoelements such as vertex sharp waves, and regular spontaneous and evoked K complexes or sleep spindles) before and after the presentation of each word. Participants underwent the transition from full wakefulness to light sleep and then oscillated primarily between the non-rapid eye movement 1 (NREM1) and NREM2 stages. Note that trials were only considered as NREM1 when there was a complete lack of alpha rhythm accompanied by sleep markers. In order to discard epochs comprising brief awakenings and microarousals (i.e., reappearance of a wake-like EEG activity for less than 3 s; percentage of trials: mean = 11.6, SD = 8) and to ensure that each trial included in the sleep conditions genuinely reflected a state of sleep, we performed an offline and conservative evaluation of sleep stages relying on strict criteria. Scoring was performed here by two trained neurophysiologists blind to experimental conditions who additionally verified that participants remained asleep after stimuli onset by tracking any electrophysiological signs of arousals (reappearance of a wake-like EEG activity for more than 3 s [13], whether the trial was associated with a button press), or any microarousals. Details and statistics about sleep scoring are provided in the Supplemental Experimental Procedures (see also Figure S1 for individual sleep architectures).

**Figure 2:**
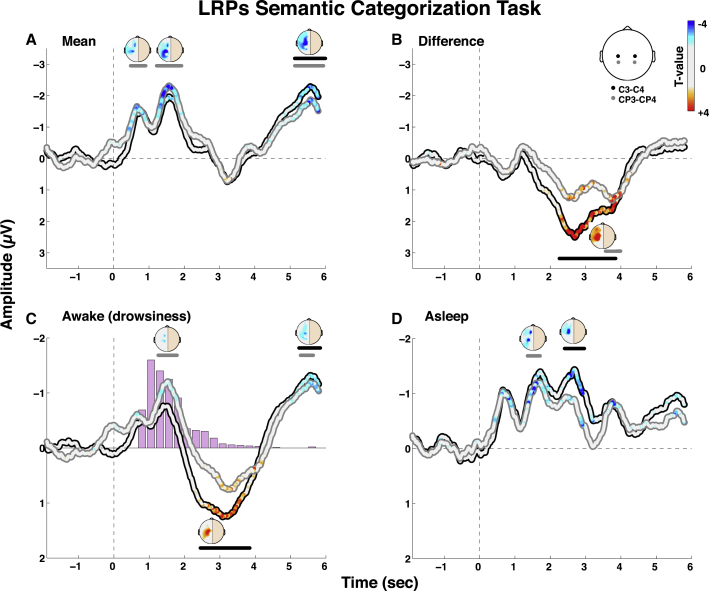
Semantic Categorization LRPs LRPs, computed by subtraction of contralateral from ipsilateral activations (see the Supplemental Experimental Procedures), revealed covert response preparation toward the target side (i.e., contralateral to the appropriate hand movement) in the vicinity of motor areas, both during wake and sleep trials. Time series show the LRP curves from stimulus onset on central (C3/C4) and central posterior (CP3/CP4) electrodes for the main effect (A), the difference between sleep and wake conditions (B), and the LRPs restricted to each condition (C and D). Bars above the time series show significant clusters with a Monte Carlo p value <0.05. 2D topographies show the LRP over the whole scalp obtained for each couple of electrodes (i.e., left/right couples) during the peak of activation of each cluster (when both electrodes pairs reached significance at the same time, the topographies were identical in both peaks, and only one is shown for brevity). The color code shows significance at the sample level (time series) and electrode level (topographies), with white color on all nonsignificant data points (p > 0.05). Histograms in the wake LRP show the RTs distribution. See also Figures S2 and S3.

LRPs constitute a direct and sensitive measure of response selection and preparation toward the target side, which is maximal in amplitude at scalp sites over the motor/premotor cortices contralateral to the responding hand [14, 15]. LRPs, traditionally computed by reference to response onset, can also be measured by reference to stimulus onset [16, 17], making them suitable to measure cortical responses in the absence of overt motor responses (i.e., during sleep). We first characterized the main (i.e., state-independent) effect of response preparation by collapsing sleep and wake trials and computing stimulus-locked LRPs using cluster-based permutation analysis (see the Supplemental Experimental Procedures). This analysis revealed a first negative deflection corresponding to the LRP, with two significant peaks at 660 and 1,620 ms, primarily over central (C3/C4) and central posterior (CP3/CP4) electrodes (Figure 2A). Interestingly, after 2,000 ms, the LRP returned to baseline for several seconds until the emergence of a second negative deflection peaking around 5,570 ms. Second, to test the difference between wake and sleep states, we subtracted the wake condition from the sleep condition (Figure 2B). Remarkably, we found no significant difference for the first LRP deflection but a clear significant effect afterward, during the opposite deflection, around 2,920 ms for C3/C4 and 3,800 ms for CP3/CP4. Restricted analysis for each vigilance state confirmed the significant early LRP deflection for wake trials and, crucially, also for sleep trials separately (Figures 2C and 2D). However, the opposite and later positive deflection was present only during wake trials. As shown in Figure 2C, the distribution of response times during wake trials suggests that the initial LRP reflects the preparation of the motor plan, while the inversion of potential appears to follow manual responses. This interpretation was confirmed by performance of a similar analysis on readiness potentials now time locked to the actual response showing the classical LRP deflection at response onset, followed immediately by the opposite deflection after the manual response (Figure S2). As discussed below, this opposite deflection in the wake condition is likely to reflect a postresponse checking mechanism that is exacerbated under conditions of drowsiness.

These results suggest that task-relevant motor preparation can be triggered during sleep. Yet, several potential issues should be addressed before drawing this conclusion. First, one might question whether participants in our study were truly asleep. Although our procedure for assessing sleep involved both online scoring and waiting for at least 2 min of absent responses before shifting to the new list of words, subjects sometimes pressed buttons either spontaneously or in response to auditory stimulation during the sleep list (14% of trials, not included in the analyses). Those button presses could be regarded as temporary arousals whereby the subjects might wake up for one or two trials, or even microarousals (i.e., less than 3 s). However, they might also reflect a nonconscious triggering of motor actions in responses to a sensory stimulation, as it is well-known in the literatures on visual masking (e.g., subliminal action priming [18]) and blindsight patients [19, 20]. In addition, past studies have shown that motor reflexes can be triggered during sleep [21]. Finally, these button presses might reflect, more simply, the fact that subjects during early sleep stages are prone to perform small movements considered in the literature as peripheral motor activations (i.e., unrelated to task or environmental contexts), such as muscle twitches [22]. Inspection of the data revealed that in most cases button presses were associated with microarousals, although there were cases where button presses were not accompanied by any signs of arousal. Importantly, we computed our sleep LRPs not only by excluding any trial with button presses, but also after performing a conservative evaluation of their vigilance state. Indeed, in order to be fully confident that the trials that we included in our analysis genuinely reflect a state of sleep, microarousals and arousals (associated with button presses or not) were detected and trials in the direct vicinity of these events were discarded, although they may be considered as sleep trials according to established guidelines. A related issue concerns the fact that our participants received the sleep list from the onset of NREM1, and thus our sleep condition reflects a mixture of NREM1 and NREM2 stages. Yet, contrary to NREM2, the NREM1 stage is sometimes regarded as an ambiguous transitory state in which awareness and responsiveness might be partially preserved [1, 7, 23]. Our data set did not allow us to reliably separate the two sleep stages due to a lack of power, as the 48 items were distributed across both stages. It thus remains possible that, even controlling for electrophysiological and behavioral markers of arousal, participants may have somehow remained conscious of the stimuli during trials scored as NREM1. To account for this potential issue and ensure that task-relevant responses can genuinely be triggered during sleep, we implemented a more stringent control of vigilance in the second experiment, where only NREM2 brain activity was considered in the sleep condition.

Another potential issue concerns the use in our study of a specific scoring method developed by Hori and collaborators for protocols with short epochs and focusing on hypnagogia [24, 25]. One might argue that this method, which is less commonly used, might underestimate the level of sleepiness and/or miss potential contaminations by microarousals in comparison to the standard scoring approach. We thus rescored our semantic decision data using the widely used guidelines of the American Academy of Sleep Medicine (AASM) [13]. We observed that the two scoring methods largely matched in terms of classifying trials in the wake or sleep state (93.1% overlap across participants; SD = 4.1%). Crucially, reanalyses of our data using the AASM scoring revealed a very similar pattern with a significant LRP deflection for sleep trials (see the Supplemental Experimental Procedures and the results in Figure S3), confirming the presence of task-relevant responses during sleep even when a more conventional method for scoring sleep was used. Finally, regarding the comparison with the wake state, a potential issue might be that the strong positive deflection that we observed with a reversal of the LRP response after an overt motor response might reflect specific conditions of drowsiness. Indeed, participants were tested while falling asleep and reaching a certain level of drowsiness, which might increase the reliance on postresponse checking mechanisms, leading to the reconfiguration and amplification/reduction of ipsi-/contralateral motor areas [26, 27]. Hence, a more direct comparison between wake and sleep states would thus not only exclude NREM1 trials as described above, but also compare sleep with conditions of full wakefulness (i.e., avoiding the drowsiness period where subjects are in the process of falling asleep).

### LRPs for Lexical Decision in Full Wakefulness and NREM2 Sleep

We performed a second experiment in which we instructed participants to perform a lexical decision on spoken material. Participants classified auditory stimuli as words versus pseudowords (i.e., items that don’t exist in the lexicon but share the same phonological properties as real words) with their left versus right hand (counterbalanced across subjects). This second experiment, in addition to dealing with the potential issues mentioned above regarding the wake-sleep transition, allowed us to verify whether the induction approach can be generalized to other classification tasks on external stimuli. Here, the nap was preceded by a session in which participants received the first list of stimuli under full wakefulness while sitting upright and not being allowed to fall asleep. Participants were then presented repeatedly with the same list while being reclined and allowed to fall asleep under similar testing conditions as in the semantic decision group. In addition, participants in this second experiment received the second list of stimuli (n = 72) only after the onset of the NREM2 stage (i.e., after the first appearance of a spontaneous K complex or sleep spindle). This design allowed contrasting LRPs during consolidated sleep versus full wakefulness rather than during the transition, where subjects are either drowsy or in a labile (NREM1) sleep stage. Since the Hori scoring is optimized for evaluating the hypnagogic period (primarily NREM1), we decided to apply AASM scoring for this second experiment while also controlling for microarousals (see the Supplemental Experimental Procedures and Figure S4 for individual hypnograms). Trials associated with a button press and microarousals dropped to 2.3% (SD = 1.7%) and 0.3% (SD = 0.6%), respectively, and were excluded from further analysis.

**Figure 3:**
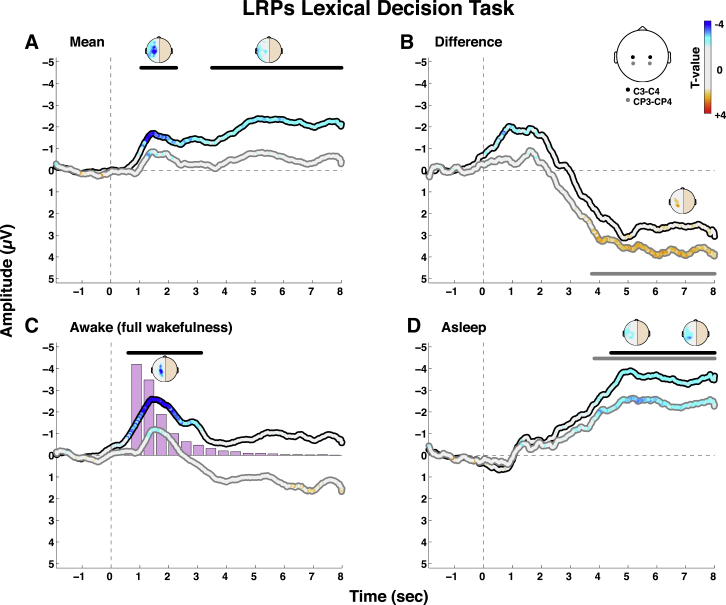
Lexical Decision LRPs See Figure 2 for a description of (A)–(D). See also Figure S2.

Analysis of the main (i.e., state-independent) effect of response preparation revealed two LRP clusters, with an early effect peaking at 1,276 ms and a later and more sustained effect peaking at 5,016 ms and extending from 3,508 ms until the end of the epoch at 8,000 ms (Figure 3A). Separate analyses for each vigilance state showed that the early LRP component was mostly driven by the wake condition, whereas the later and more sustained cluster was primarily driven by the sleep condition (Figures 3C and 3D). Indeed, whereas the LRP in the wake condition was rather transient and overlapped with the reaction times distribution, the LRP during sleep corresponded to a large and sustained response developing slowly over time. As a consequence, the contrast of wakefulness (i.e., the difference between wake and sleep trials; Figure 3B) revealed a trend for an early negativity, suggesting a stronger early LRP under wake conditions, and a significant and sustained positivity for the late component, reflecting a delayed LRP during sleep. These results confirm the presence of covert task-relevant responses to speech during sleep and extend the finding in the semantic decision from experiment 1 to the classification of lexical properties during the NREM2 state. Notably, the opposite deflection found in experiment 1 under drowsy conditions was not observed here under conditions of full wakefulness. It is also interesting to observe that the LRP during sleep was further delayed in time compared to experiment 1. We interpreted this finding as the involvement of slower mechanisms of evidence accumulation during the N2 stages in experiment 2, compared to the mixture of N1 and N2 responses in experiment 1 [1].

**Figure 4:**
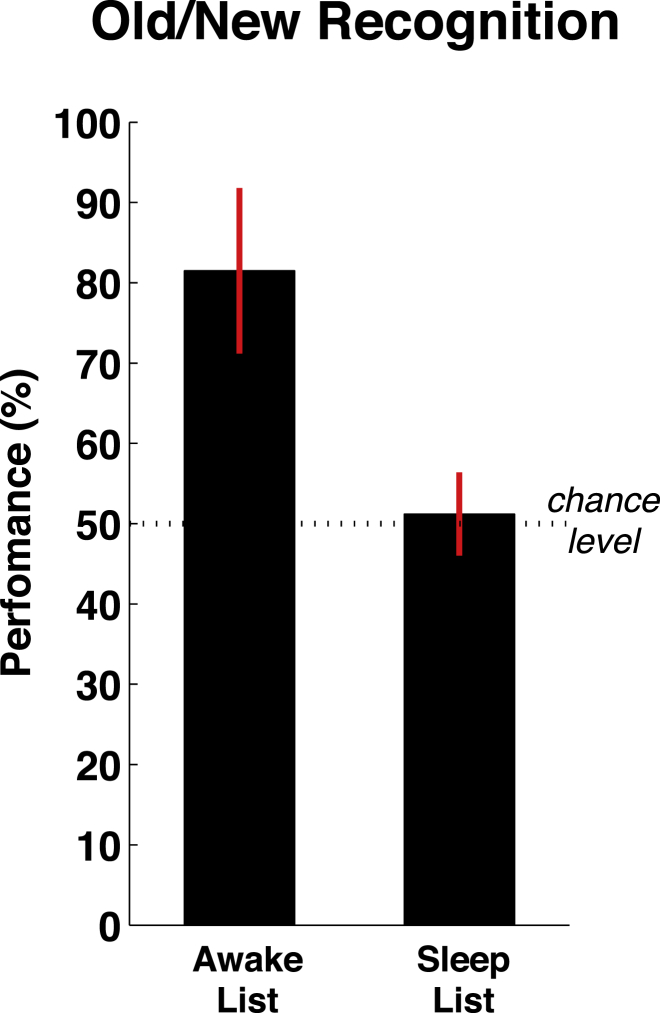
Results of the Old/New Explicit Recognition Test Performed Immediately after the Nap Participants received stimuli from the wake list, sleep list, and an entirely new list and were instructed to indicate which items had been played previously or were entirely new. Performance, computed by comparison of responses of the wake and sleep lists to the new list, revealed high-accuracy performance for the wake list but chance-level performance for stimuli presented during sleep. Error bars indicate 1 SD.

One might still argue that participants in our study were somewhat aware of the spoken stimuli, with fleeting microarousals that are difficult to detect in the EEG, resulting in a state of transient arousal/drowsiness not allowing them to perform an overt behavioral response. In order to directly address this issue, through an operational measure of stimulus awareness, we instructed participants to perform an explicit recognition task right after the lexical decision experiment, after regaining full consciousness. They were presented with the stimuli from the wake list, from the sleep list, or from a new list of completely novel items (counterbalanced across participants) and were instructed to classify each stimulus as either old or new and then rate their confidence about their decision on a scale ranging from 1 (completely guessing) to 7 (completely sure). Results of the posttest (see Figure 4) revealed that participants could distinguish new words from words presented during the wake period (performance = 81.5%, *d′* = 2.16, both p < 0.0001) but, crucially, not from words presented during sleep (performance = 51.2%, *d′* = 0.13, both nonsignificant [n.s.]). Consistently, the postdecision confidence estimates also did not differ between the new and sleep lists (mean confidence = 4.79 versus 4.80, respectively; n.s.), whereas they were significantly higher for the list presented during the preceding period of wakefulness (5.80, both p < 0.001). Overall, these results add strong evidence supporting the fact that participants did not have explicit access to the stimuli presented during sleep and confirm that the LRP obtained during sleep most likely reflects a nonconscious form of speech processing.

## Discussion

There is now converging evidence that environmental stimuli can still be processed during sleep, at least to a certain degree [2]. For instance, meaningful stimuli (e.g., own names, own baby’s cry, and fire alarm) are more likely to lead to awakening [28, 29, 30, 31]. Furthermore, sleeping participants, while in REM or NREM stages, can create novel sensory associations between tones and odors [3] or reactivate existing semantic associations as evidenced by the presence of an N400 component in EEG [4, 5, 6, 7]. Besides, sleepwalkers are able to re-enact recently learned sequences of movements [32]. Thus, there is evidence, albeit scarce, that sleep does not preclude meaning extraction or the activation of learned associations and sensorimotor mappings. However, to date, no study has directly tested the possibility that environmental stimuli are processed in a flexible manner, all the way up to the preparation of task-relevant responses. Here, using LRPs, we show that sleeping participants are still able to prepare for the appropriate response on semantic and lexical decision tasks practiced before falling asleep. The current design, using single-word presentations, does not directly test for meaning extraction (unlike classical N400 paradigms using word pairs or sentences). However, our study reveals speech processing through semantic and lexical categorization by demonstrating the preparation of motor plans conditional on the meaning of spoken words. These results not only confirm previous findings showing that semantic information can still be extracted during sleep, but further show that this nonconscious meaning extraction can be routed by the task context and reach higher processing levels, up to motor preparation stages. This suggests that when processing environmental information during sleep, at least during early NREM stages, only the final stages related to action execution might be suppressed.

An important remaining question, therefore, is where in the neural stream ranging from motor preparation to action execution lays the bottleneck responsible for the lack of behavioral responses. Previous studies revealed that sleep is associated with both the inhibition of dorsolateral prefrontal cortex, a crucial area for executive functions [8, 9], and the functional breakdown in thalamocortical connectivity, associated with the loss of wakefulness and sensory awareness [33]. On the contrary, neural activity in other cortical regions, including sensorimotor areas, does not importantly differ from the wake stage [9, 34, 35]. The preserved functionality of these regions may support elaborate—albeit automatized—cognitive processes such as those observed in the present study. One might even expect that, as long as a given task has been induced during the wake stage, almost any processing stream could potentially remain activated during sleep. Future studies will be necessary to address this issue and, in particular, whether even higher-order regions dealing with executive functions such as cognitive control or task switching can be triggered using a task-induction strategy.

It remains to be elucidated whether this finding would generalize to other sleep stages, and in particular to REM sleep, in which there is an almost complete muscular paralysis but electrophysiological activity is closer to that of wakefulness. On the one side, because the strong inhibition of motor neurons during REM sleep involves only subcortical structures (such as the locus coeruleus, which targets motor neurons in the spinal cord), and given the relatively preserved information processing capabilities during this stage [36], one might still expect similar covert responses as found here. On the other side, these findings might be restricted to the initial stages of sleep, during which the thalamus is mostly deactivated whereas large parts of the cortex remain active [37]. Future studies relying on full-night protocols will be necessary to address whether the integration of semantic and decision processes can bypass early sleep stages.

Beyond revealing unsuspected processing capabilities in the sleeping brain, this study uncovers a promising avenue to study nonconscious processes. Research investigating the distinction between conscious and nonconscious mechanisms (the so-called “contrastive approach” [38]) generally focuses on the notion of contents of consciousness. In this framework, the participant can be nonconscious “of” a specific content as in a typical situation of visual masking but remains fully conscious in the intransitive sense of being aroused and vigilant. For instance, although previous studies using subliminal priming have shown that invisible primes can trigger lateralized readiness potentials [10, 39], participants in these studies were still having conscious access to their goal-directed behaviors in order to perform a specific task on target stimuli. Here, although sleeping participants may continue to process information in a goal-oriented manner, this task set is presumably maintained without the participant being conscious of it. Moreover, our experimental approach relying on levels rather than contents of consciousness not only allows examination of the neural consequences of perceptual processes when the subject is nonconscious in any possible respects, but also offers the opportunity to use sensory stimuli that are not degraded in any manner. Indeed, the strong degradation of sensory signals typically used to achieve robust unawareness in masking studies, either in the visual [11] or the auditory modality [40], unavoidably decreases the strength of neural responses, especially in brain regions dealing with high-level information [11]. Hence, studying sleep in this context allows pushing further the limits and extents of nonconscious processes and establishing the properties of a broader and more natural type of cognitive unconscious.

## Supplemental Information

Document S1. Supplemental Experimental Procedures and Figures S1–S4

Document S2. Article plus Supplemental Information
