# Supplementary material for: Inducing Task-Relevant Responses to Speech in the Sleeping Brain
Source: Curr Biol. 2014 Sep 22;24(18):2208–14. doi: 10.1016/j.cub.2014.08.016 (PMC4175175; doi:10.1016/j.cub.2014.08.016)
Supplement: Document S1. Supplemental Experimental Procedures and Figures S1–S4 [file mmc1.pdf]

Current Biology, Volume *24*

Supplemental Information

Inducing Task-Relevant Responses
to Speech in the Sleeping Brain

Sid Kouider, Thomas Andrillon, Leonardo S. Barbosa, Louise Goupil, and Tristan A. Bekinschtein

Supplemental data

Figure S1. *Top*. Average power spectra across participants for wake trials (blue) and sleep trials (red) in Experiment 1. Note the turnaround between alpha (8-13 Hz) and beta (20-40 Hz) rhythms predominant in wakefulness and sleep-related oscillations (delta (0.1-4 Hz), theta (4-7 Hz), spindle range (11-16 Hz)). Power spectra were computed on C3/C4 referenced to the mastoids with a fast Fourier transform and averaged across subjects. *Purple bars* mark frequencies for which power was significantly higher in sleep compared to wake (paired t-test, 0.05, false detection rate correction). *Light green bars* mark frequencies for which power was significantly higher in wake compared to sleep (paired t-test, 0.05, false detection rate correction). Red and blue shadings denote standard-error to the mean. ***Bottom*. Individual hypnograms.** *Black lines* show the vigilance state per trial visually scored using AASM guidelines. *Grey dots* show recorded response times (RTs). Note the large variability in RTs typical of drowsiness. The line below each hypnogram contains information about the stimulus list (*cyan*: wake list; *magenta*: sleep list) and the line above each hypnogram depicts the final scoring taking into account behavioral and electrophysiological criteria (*blue*: “wake” trials ; *red*: “sleep” trials). Related to Figure 1.

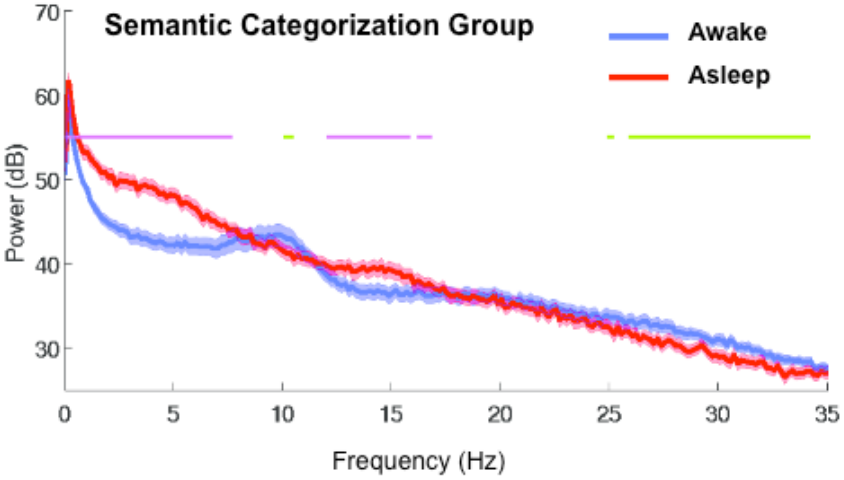
Semantic Categorization Group
Awake
Asleep
Power (dB)
Frequency (Hz)
70
60
50
40
30
0
5
10
15
20
25
30
35

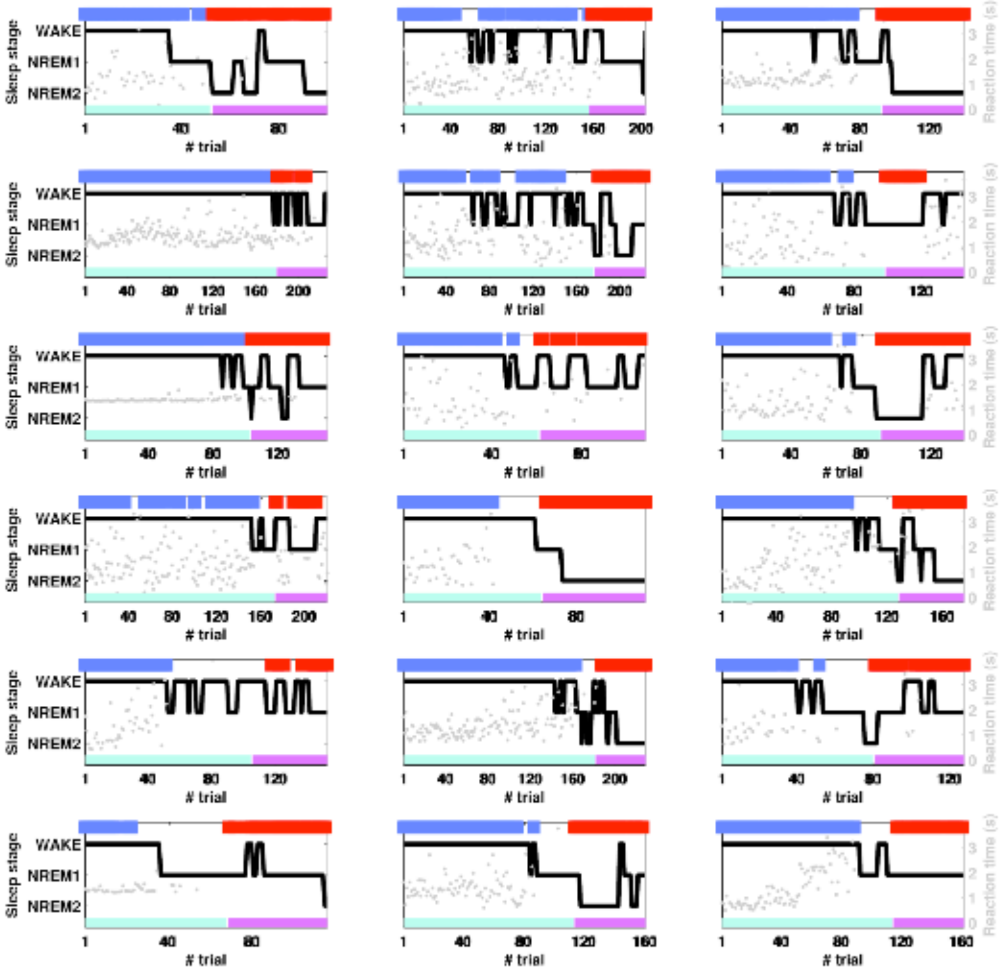
Sleep stage
WAKE
NREM1
NREM2
trial
Reaction time (s)

Figure S2. Response-locked LRPs for Experiment 1 (top panel) and Experiment 2 (bottom panel). LRPs were here averaged with respect to the participant response on each trial (i.e. 0 ms corresponds to the response time). Baseline correction was performed with respect to a -4000 to -2000ms period before stimulus onset. Time-series show the LRP curves on central (C3/C4) and central posterior (CP3/CP4) electrodes (See Figure 2 for more details). Related to Figure 2 and Figure 3.

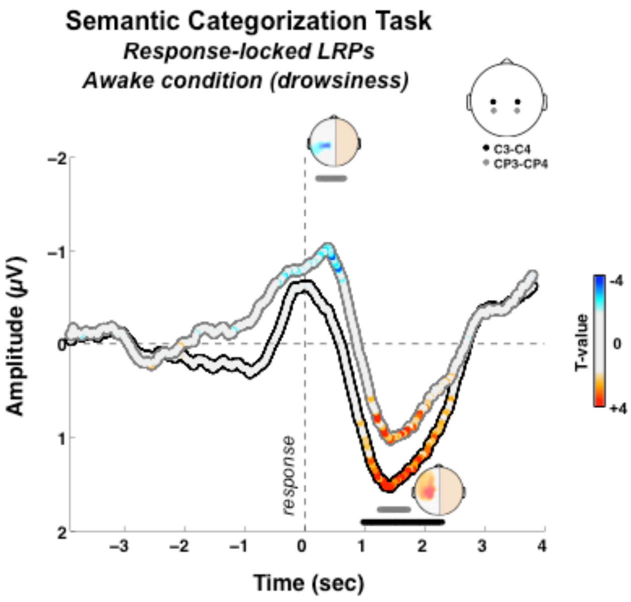

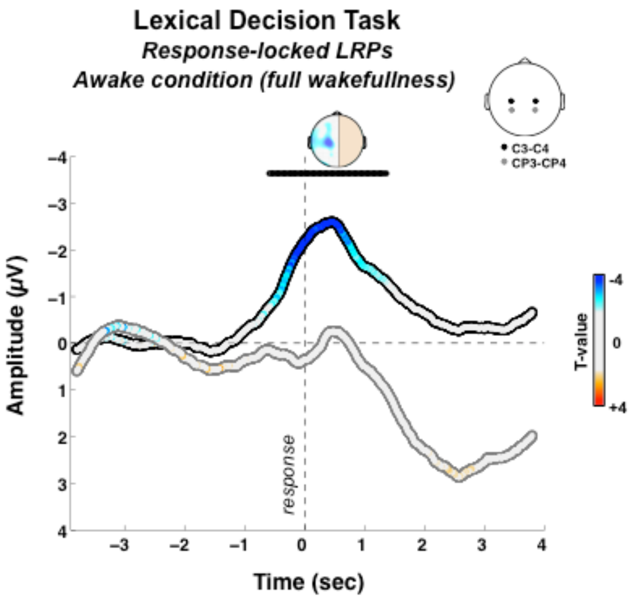

Figure S3. Stimulus-locked LRPs in Experiment 1 with standard sleep scoring. LRPs for the sleep condition in which trials were scored according to standard guidelines (see section on "Supplemental sleep scoring using standard guidelines" and Figure 2 for more details). Related to Figure 2.

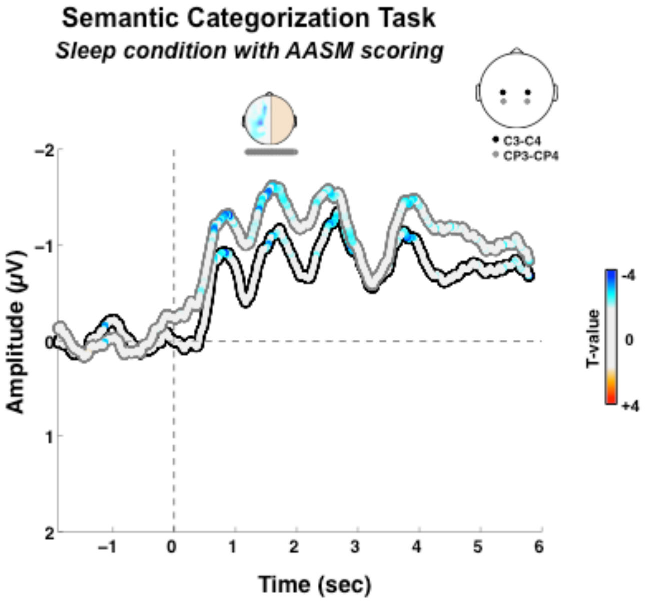

Figure S4. ***Top.*** **Average power spectra across participants for wake trials (blue) and sleep trials (red) in Experiment 2.** ***Bottom*****. Individual hypnograms** See Figure S1 for details. Related to Figure 1.

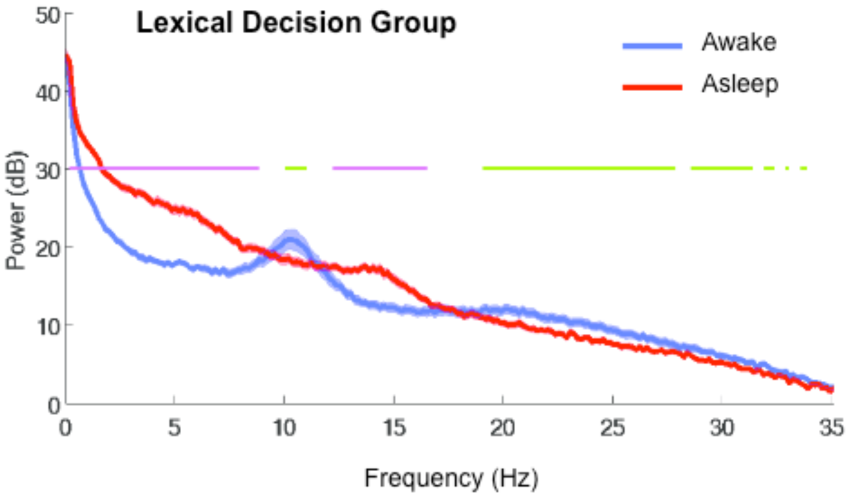

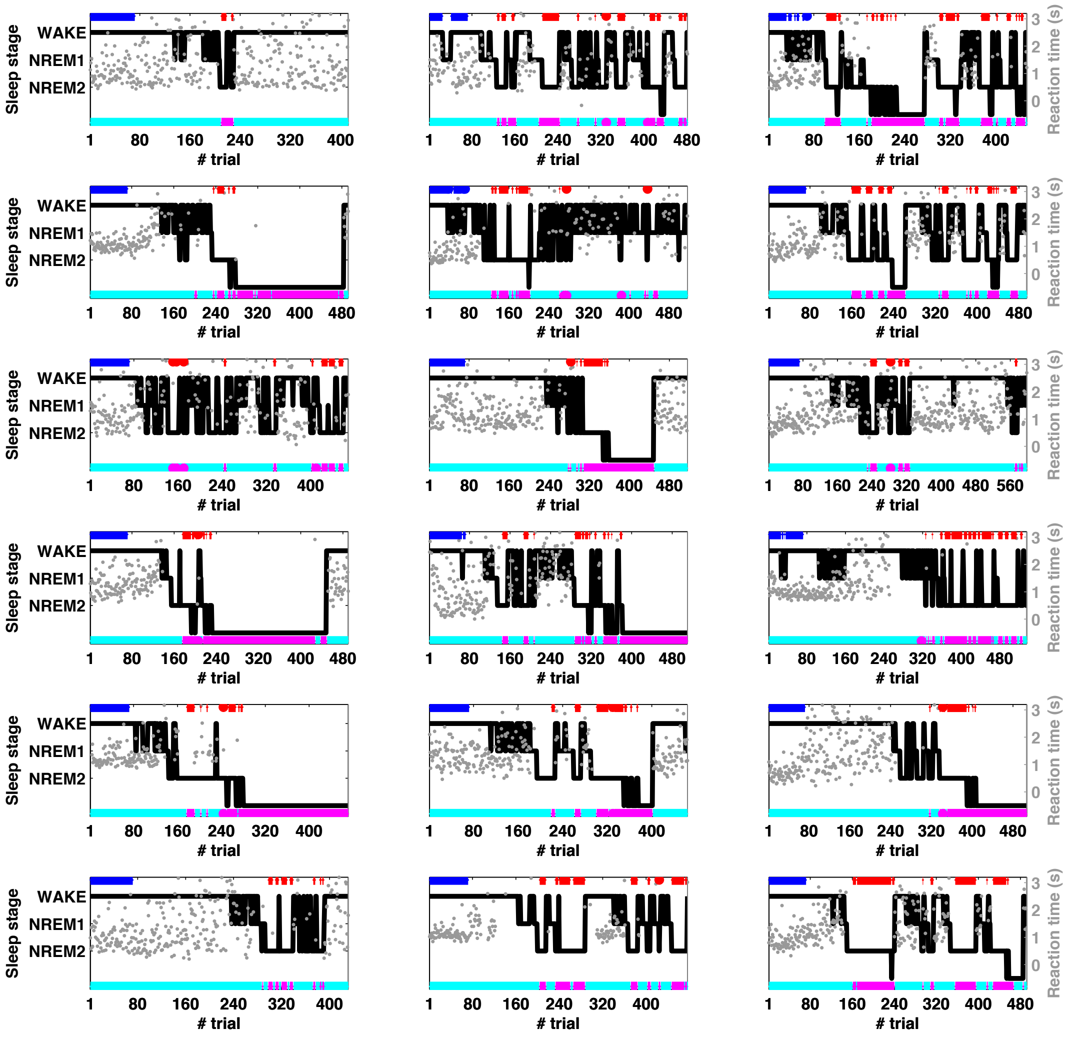

Sleep stage
WAKE
NREM1
NREM2
trial
Reaction time (s)
0 1 2 3
1 80 160 240 320 400 480 560

Supplemental Experimental Procedures

Participants. Eighteen native English speakers (6 women and 12 men, age range: 18-30 years) took part in Experiment 1. An additional 29 participants were tested but not included in the final analysis because of a failure to fall asleep (N=27) or due to excessive artefacts in the EEG signal (N=2). For Experiment 2, 18 native French speakers (12 women and 4 men, age range: 20-28 years) were included out of 22 subjects. Four subjects were thus excluded either due to not falling asleep (N=1) or note reaching the N2 stages (N=3). All subjects were right-handed, and reported no auditory, neurological or psychiatric alterations. To increase the probability that participants would fall asleep in our experimental setup, only easy sleepers, as assessed by the Epworth Sleepiness Scale, were selected for this study. This scale evaluates whether participants are used to easily falling asleep, for instance when watching TV or during train trips. Recruited participants were considered healthy with relatively high ESS scores but not corresponding to a condition of pathological sleep such as hypersomnia :the average ESS scores were 10.4 with (range 7-14) for Experiment 1, and 11.6 (range 7-16) for Experiment 2, while the maximum possible score is 24. Participants were also asked to avoid exciting substances as coffee, and to sleep 1-2 hours (20%) less than usual the night preceding experiment 1 and 2-3 hours (30%) less than usual for Experiment 2. They signed a written consent and were paid for their participation. Both experiments were approved by the relevant local ethical committees (Cambridge psychology research ethics committee for Experiment 1, Conseil d'évaluation éthique pour les recherches en santé for Experiment 2).

Stimuli. For Experiment 1, stimuli were spoken words selected from the CELEX lexical database (Linguistic Data Consortium, University of Pennsylvania). There were 48 names of objects and 48 names of animals. Half were monosyllabic and the other half disyllabic, with animal and object names matched as closely as possible in terms of combined (spoken and written) log lemma frequencies, as confirmed by an independent t-test ($p > 0.10$). Additionally, words within the two categories were matched in a pair-wise fashion regarding their phonological properties: each object name was matched with a similar animal name (for example “quilt” was matched with “quail”),

ensuring that animal and objects names could not be differentiated in terms of sub-semantic (i.e., phonological) properties. The words were tape-recorded by a female voice and digitized. Two lists of 48 stimuli each were produced, one for the wake period and the other for the sleeping period (counterbalanced across participants). For Experiment 2, the material consisted of 216 auditory stimuli corresponding to 108 pairs of words and pseudowords (half CVC monosyllabic and half CV-CV disyllabic) recorded by a male native French speaker and digitized. Within each pair, words and pseudowords were matched in length and phonological (consonant-vowel) structure. The words were selected from the Lexique database [S1] and the pseudowords were all legal and pronounceable combinations of sounds in French. Three lists of 72 stimuli matched for frequency and phonological structure were constructed such as to be counterbalanced across participants for the wake period, the sleep period and the new list in the old/new recognition task following the main experiment.

Procedure. Participants were lying down with their eyes closed in a comfortable reclining chair in a dark and electrically and acoustically shielded EEG cabin. Stimuli were presented binaurally through headphones (Experiment 1) or through loud speakers (Experiment 2). Participants were instructed to perform a semantic categorization on whether each spoken word referred to an animal or to an object (Experiment 1) or to perform a lexical decision on whether each spoken stimulus existed or not in French (Experiment 2), by pressing a button with either their left or right hand (with response hand counterbalanced between participants). For Experiment 1, they were told that they could fall asleep at any time during the task, but were asked not to stop responding deliberately before falling asleep (i.e. not to stop responding in order to fall asleep). For Experiment 2, participants first performed a full session with the wake list items (about 10 minutes) under conditions were they were fully wake and not allowed to fall asleep, before hearing the wake list again while being reclined and allowed to fall asleep under similar testing conditions as in Experiment 1. Testing conditions encouraged the transition towards sleep while remaining engaged with the same task-set (explicit allowance to fall asleep, dark room, eyes closed, reclining chair, several repetitions of the first stimulus list, long inter stimulus interval). The continuous, uninterrupted flow within and across the two lists of stimuli was aimed at reducing the probability of awakening.

While being awake, participants could hear up to 4 repetitions of the first list. In Experiment 1, they were presented with the second list of 48 items only once during sleep while in Experiment 2, they could receive the second list of 72 items up to 3 times to increase the number of sleep trials. Stimuli were presented in a random order with an inter-stimulus interval varying between 6 and 9 seconds in Experiment 1 and a fixed duration of 9 seconds for Experiment 2. The presentation of spoken items would switch to the second list without interrupting the pace of the experiment whenever the participant was assessed by the experimenter as being asleep (Experiment 1) or as entering the NREM2 stage (Experiment 2, see details below), For Experiment 2, stimulation was switched back to the wake list in cases of return to NREM1, (micro)-awakenings and/or button presses. Stimulus delivery and response collection was controlled by the E-Prime software (Psychology Software Tools, Pittsburgh, PA) for Experiment 1 and by the Matlab (MathWorks Inc. Natick, MA, USA) using the Psychophysics Toolbox [S2] for Experiment 2.

EEG recordings and analysis. The electroencephalogram was continuously recorded from 64 Ag/AgCl electrodes (NeuroScan Labs system for Experiment 1; Electrical Gegodesic Inc system for Experiment 2), with Cz as a reference. The impedance for electrodes was kept following constructor recommendations. Data were acquired with a sampling rate of 500 Hz (Experiment 1) or 250 Hz (Experiment 2). For the wake trials, only the first list occurrence was analysed (Experiment 1: N=48, Experiment 2: N=72). Continuous data were epoched from -2000 to 6000ms (Experiment 1) or to 8000ms (Experiment 2) in relation to stimulus onset, low-pass filtered at 30Hz and baseline corrected in respect to the pre-stimulus window of 2000ms. Trials with any electrode passing an absolute threshold (Experiment 1 with the NeuroScan system: 1000 μV, Experiment 2 with the Electrical Gegodesic Inc system: 250 μV) were rejected from the analysis (this concerned only non-physiological events). We used a very liberal threshold because sleep trials may contain large-magnitude K-complexes.

Separate averages were computed for left (L) and right (R) hand trials, resulting in two average waveforms for each electrode and participant. Stimulus locked LRP were then computed according to the procedure by Coles (1989, [S3]), using the ERP waveforms recorded from corresponding electrode pairs in each hemisphere as follow:

LRP = [(R hand – L hand trials) on L electrode + (L hand – R hand trials) on R electrode] * 0.5

Statistical significance was assessed through cluster/permutation statistics calculated within participants, allowing us to deal with the potential issue of multiple comparisons in a principled manner. Each cluster was constituted by the samples that consecutively passed a specified threshold (in this case sample p-value of 0.1). As demonstrated by Maris & Oostenveld (2007, [S4]), this threshold doesn't change the type-1 error, and the method controls for false alarms independent of this value. The cluster statistics was chosen as the sum of the t-values of all the samples in the cluster. Then, we compared the cluster statistics of each cluster with the maximum cluster statistics of 1000 random permutations. The significance of LRPs was assessed during both for the wake and sleep conditions by using a threshold monte-carlo p-value of 0.05.

Sleep assessment for Experiment 1. Sleep onset was determined *online* by relying on both behavioural and electrophysiological criteria. Participants were assumed to be asleep if they were not responding for at least 2 minutes, and if they were presenting EEG and EOG patterns characteristic of NREM sleep: reduction of fast rhythms (alpha – beta) in favour of slower rhythms (theta waves), slow-eye movements, vertex sharp waves and possibly evoked and/or spontaneous K-complexes and sleep spindles. Once sleep onset was confirmed, the first list was switched to a second one, never heard by the participant. For Experiment 1, after switching list, participants could occasionally press a button (14% of the trials in the sleep list). An offline sleep assessment was therefore conducted to confirm the sleeping state and to remove arousals or ambiguous trials (i.e., with potential micro-arousals), as well as trials with a button press. For Experiment 1, in which we concentrated on wake-to-sleep transition, we used an extension of standard sleep staging adapted and validated by Hori and

collaborators [S5, S6]. This method allows for a more refined sleep scoring since it uses smaller epochs prior to the stimulus onset (4 seconds) and allows for a more detailed characterisation of the hypnagogic period at the time of the auditory stimulation. Wakefulness was characterized by regular responses to stimuli, presence of fast low-amplitude rhythms such as alpha rhythms (8-13 Hz) especially on occipital electrodes, eye-blinks or saccades. Participants were declared asleep after the disappearance of alpha rhythm, replaced by slower oscillations (vertex sharp waves, theta rhythms). On the EOG, presence of slow eye movements was also indicative of the wake to NREM1 transition. Finally, when spontaneous K-complexes or spindles occurred in the 4s epoch prior to stimulus onset, the trial was scored as NREM2.

Importantly, in our protocol, it was crucial to assess not only the context in which stimuli were played (determined through the careful examination of the pre-stimulus activity) but also how these stimuli affected brain activity by potentially triggering micro-arousals. In order to retain as sleep trials only those for which participants were genuinely asleep and remained in this state, we visually detected and marked every sign of arousal (increase in low-amplitude fast rhythms such as alpha oscillations or oscillations above 16Hz for more than 3 seconds and stable for at least 10 seconds) or micro-arousal (increase in low-amplitude fast rhythms such as alpha oscillations or oscillations above 16Hz for less than 3 seconds) following the stimulus onset (see S8). Although micro-arousals were accompanied with behavioural responses in only a few cases, such trials were discarded from our analysis to ensure a conservative sleep scoring. This resulted in a total average of 70.8 trials per participant in this experiment, corresponding to 42.6 and 28.2 trials per participants in the wake and sleep conditions, respectively. Remaining trials were discarded (e.g. trials from the sleep list that were potentially associated with micro-arousals and/or with a button press). Among the trials included in the sleep condition, 79.4% were scored as NREM1 and 19.7% as NREM2. However, in order to satisfy standard definitions, NREM2 was scored only after the first occurrence of a *spontaneous* spindle or K-complex. As a consequence, evoked K-complexes or sleep spindles were still observed in 27.2% of NREM1, which reflects a deeper sleep stage than the standard NREM1. None of the participants reached the NREM3 stage or showed a REM episode. When considering a -2 to 4s

window around stimuli onset, K-complexes were observed in 24.5% of sleep trials (23.2% of NREM1 trials) and sleep spindles in 8.5% of sleep trials (4.9% of NREM1 trials).

Note that no consensus exists for a simple (e.g. scalar) criterion that can be used automatically to separate sleep from wake trials, arguably because of the individual differences in terms of amplitude/frequency range used to score vigilance states (see for instance [S7] for alpha and theta rhythms). For these reasons, the sleep assessment was performed by visual inspection, ensuring an evaluation that is both conservative (i.e., eliminating any sign of micro-arousal) and adaptative (i.e., taking into account individual variability). Nevertheless, to verify the validity of our sleep scoring methodology, we developed a scalar criterion that would constitute a quantitative evaluation of the difference between trials in the sleep and wake conditions. This scalar Vigilance Index (VI) was defined as the ratio of the mean power in specific frequency ranges computed on C3-C4 electrodes over each epoch (i.e., -2 to 6 seconds around stimulus onset), using a fast Fourier transform:

VI = [delta power + theta power + spindle power] / [alpha power + high-beta power]

With delta corresponding to 0.1 – 4 Hz, theta to 4 – 7 Hz, spindle frequency to 11 – 16 Hz, alpha to 8 – 13 Hz, and high-beta to 20 – 40 Hz). Low-Beta was not included as it overlaps with the frequency of spindles. For each epoch, power was normalized by the power in high frequency range (215 – 245 Hz). Delta, theta and spindle power being classically associated with sleep while alpha and high-beta are associated with wakefulness (see Figure S1 Panel B for an illustration), "sleep" trials should show higher VI values than "wake" trials. VI was computed for every trial in the sleep and wake conditions. The distribution of VI values across all trials was bi-modal ($p<0.01$, Hardigan Dip Test). Importantly, when considering VI values for "sleep" and "wake" trials separately, we checked that their respective distributions were statistically different ($p<0.001$, unpaired t-test). This was also true when considering subjects individually ($p<0.001$, unpaired t-test, Bonferroni correction). This demonstrates that we are genuinely dealing with two distinct brain states in our study.

Supplemental sleep scoring of Experiment 1 using standard guidelines. To ensure that our results did not reflect an underestimation of the level of sleepiness due to the sleep scoring method we used, and thus the possibility of missing potential contaminations by micro-arousals, we performed a re-scoring of our data using standard guidelines of the AASM [8]. Data were first continuously scored as wake, NREM1 and NREM2 using 20s epochs. Regular correct responses to stimuli, presence of alpha rhythms on occipital regions, eye-blinks or saccades were indicative of wakefulness. NREM1 was defined by the replacement of alpha rhythms with theta rhythms. Presence of slow eye movements, vertex sharp waves, evoked K-complexes or sleep spindles were also indicative of NREM1 onset. Finally, epochs showing spontaneous K-complexes or spindles were scored as NREM2. In order to retain as sleep trials only trials for which participants were and remained asleep, NREM1 and NREM2 trials associated with motor responses or micro-arousal (increase in low-amplitude fast rhythms lasting less than 3s) and arousals (e.g. associated or not with button presses) were discarded from further analysis. The corresponding LRP results are presented in figure S3.

Sleep assessment for Experiment 2. For Experiment 2, in which we directly compared a state of full alertness with NREM2, we relied exclusively on the standard sleep scoring method of the ASSM relying on 20-30 seconds epochs [S8] which is more adapted to the evaluation of deeper sleep states. In order to focus on NREM2, participants were assumed to be fully asleep if they were unresponsive and after the occurrence of the first spontaneous K-complex or sleep spindle (i.e. not appearing within at least 1 second follwing stimulus onset). There were also trials scored as NREM3 but those were not included in the final analysis as it concerned fewer trials and only a restricted set of participants (N=11). There was a total average of 147.7 trials per participant, corresponding to 68.7 and 79 trials in the wake and sleep conditions, respectively. The same procedure as for Experiment 1 was used here to discard trials micro-arousals and button presses.

Old/new recognition post-test. Experiment 2 was followed, after awakening, by an explicit recognition test in which they were presented, in random order, with spoken words that were

previously presented during the wake or sleep period, or new words that were not presented before. They were instructed to report, using one of two keys on a keyboard, whether the word was old (i.e., presented in the wake or sleep list) or new, without time pressure. Following their answer, they indicated their level of confidence on a scale ranging from 1 (completely guessing) to 7 (completely sure), again without time pressure. Each participant was presented with 108 words (36 words per condition). The old items from the sleep conditions that were subsequently scored as reflecting N1, (micro)-awakenings and/or button presses were discarded from the analysis, to match items used in the LRP analysis.

Supplemental references

S1. New, B., Pallier, C., Brysbaert, M., and Ferrand, L. (2004). Lexique 2: a new French lexical database. Behavior research methods, instruments, & computers : a journal of the Psychonomic Society, Inc *36*, 516-524.

S2. Brainard, D.H. (1997). The Psychophysics Toolbox. Spat Vis *10*, 433-436.

S3. Coles, M.G. (1989). Modern mind-brain reading: psychophysiology, physiology, and cognition. Psychophysiology *26*, 251-269.

S4. Maris, E., and Oostenveld, R. (2007). Nonparametric statistical testing of EEG- and MEG-data. Journal of neuroscience methods *164*, 177-190.

S5. Hori, T., Hayashi, M., and Morikawa, T. (1994). Topographical EEG changes and the hypnagogic experience. . Sleep Onset: Normal and Abnormal Processes, 237– 253.

S6. Tanaka, H., Hayashi, M., and Hori, T. (1997). Topographical characteristics and principal component structure of the hypnagogic EEG. Sleep *20*, 523-534.

S7. Klimesch, W., Doppelmayr, M., Schwaiger, J., Auinger, P., and Winkler, T. (1999). 'Paradoxical' alpha synchronization in a memory task. Brain Res Cogn Brain Res *7*, 493-501.

S8. Iber, C., Ancoli-Israel, S., Chesson, A., and Quan, S.F. (2007). The AASM Manual for the Scoring of Sleep and Associated Events: Rules, Terminology and Technical Specifications, (Westchester, IL: American Academy of Sleep Medicine).
