# Supplementary material for: Inducing Task-Relevant Responses to Speech in the Sleeping Brain
Source: Curr Biol. 2014 Sep 22;24(18):2208–14. doi: 10.1016/j.cub.2014.08.016 (PMC4175175; doi:10.1016/j.cub.2014.08.016)
Supplement: Document S2. Article plus Supplemental Information [file mmc2.pdf]

Current Biology 24, 2208–2214, September 22, 2014 ©2014 The Authors http://dx.doi.org/10.1016/j.cub.2014.08.016

Report

Inducing Task-Relevant Responses to Speech in the Sleeping Brain

Sid Kouider,[1,*] Thomas Andrillon,[1,2] Leonardo S. Barbosa,[1,2] Louise Goupil,[1,2] and Tristan A. Bekinschtein[3,4]
[1]Laboratoire de Sciences Cognitives et Psycholinguistique, CNRS/EHESS/DEC-ENS, 29 Rue d'Ulm, 75005 Paris, France
[2]Ecole Doctorale Cerveau-Cognition-Comportement, Université Pierre et Marie Curie, 9 Quai Saint Bernard, 75005 Paris, France
[3]Cognition and Brain Sciences Unit, Medical Research Council, 15 Chaucer Road, Cambridge CB2 7EF, UK
[4]Department of Psychology, University of Cambridge, Downing Street, Cambridge CB2 3EB, UK

Summary

Falling asleep leads to a loss of sensory awareness and to the inability to interact with the environment [1]. While this was traditionally thought as a consequence of the brain shutting down to external inputs, it is now acknowledged that incoming stimuli can still be processed, at least to some extent, during sleep [2]. For instance, sleeping participants can create novel sensory associations between tones and odors [3] or reactivate existing semantic associations, as evidenced by event-related potentials [4–7]. Yet, the extent to which the brain continues to process external stimuli remains largely unknown. In particular, it remains unclear whether sensory information can be processed in a flexible and task-dependent manner by the sleeping brain, all the way up to the preparation of relevant actions. Here, using semantic categorization and lexical decision tasks, we studied task-relevant responses triggered by spoken stimuli in the sleeping brain. Awake participants classified words as either animals or objects (experiment 1) or as either words or pseudowords (experiment 2) by pressing a button with their right or left hand, while transitioning toward sleep. The lateralized readiness potential (LRP), an electrophysiological index of response preparation, revealed that task-specific preparatory responses are preserved during sleep. These findings demonstrate that despite the absence of awareness and behavioral responsiveness, sleepers can still extract task-relevant information from external stimuli and covertly prepare for appropriate motor responses.

Results

We studied whether the categorization of spoken words can still trigger task-relevant motor plans during early sleep stages. One main difficulty in addressing this issue consists in instructing a new task to sleeping subjects, arguably because prefrontal regions dealing with executive functions are then particularly suppressed in comparison to other cortical regions [8, 9]. One potential solution is to rely on the induction approach commonly used by studies on implicit perception in awake participants. This research reveals that the processing stream involved in making a semantic classification can, through explicit practice, be automatized and bypass prefrontal regions. Under those conditions, the categorization of visual words and numbers can lead to the covert activation of motor cortex even when those stimuli are masked and presented below the threshold of consciousness [10, 11]. In the current study, we extend this task induction strategy to track the ability of sleepers in extracting task-relevant information from speech and preparing for the appropriate motor plan.

LRPs Reveal Semantic Classification and Response Preparation before and after Falling Asleep

We recorded the electroencephalogram (EEG) of human participants while they were awake and instructed them to classify spoken words as animals or objects (Figure 1). This procedure allowed us to compute lateralized readiness potentials (LRPs)—a neural marker of response selection and preparation [12]—by mapping each specific semantic category to a specific motor plan (e.g., animals with the right hand and objects with the left hand, counterbalanced across participants). This design allows for the assessment of lateralized response preparation toward the side associated with the appropriate semantic category. Thus, it allows for testing of whether sensory signals are processed beyond semantic levels by probing how the meaning extracted from external words can lead to the covert selection and preparation of context-dependent actions. Testing conditions encouraged the transition toward sleep while remaining engaged with the same task set: subjects received explicit allowance to fall asleep and were sitting in a dark room, eyes closed, in a reclining chair, listening to several repetitions of the same list of stimuli with a long intertrial interval of 6–9 s. Crucially, participants received an entirely new list of words (n = 48) during sleep to ensure that their responses were based on the extraction of word meaning rather than a mere reactivation of stimulus-response associations established during the wake stage.

Sleep onset was assessed online both behaviorally, by ensuring the absence of overt responses for at least 2 min of stimulation, and electrophysiologically, through sleep markers (i.e., disappearance of low-amplitude alpha/beta rhythms and development of high-amplitude delta/theta rhythms [see Figure S1 available online], presence of slow eye movements and other sleep graphoelements such as vertex sharp waves, and regular spontaneous and evoked K complexes or sleep spindles) before and after the presentation of each word. Participants underwent the transition from full wakefulness to light sleep and then oscillated primarily between the non-rapid eye movement 1 (NREM1) and NREM2 stages. Note that trials were only considered as NREM1 when there was a complete lack of alpha rhythm accompanied by sleep markers. In order to discard epochs comprising brief awakenings and microarousals (i.e., reappearance of a wake-like EEG activity for less than 3 s; percentage of trials: mean = 11.6, SD = 8) and to ensure that each trial included in the sleep conditions genuinely reflected a state of sleep, we performed an offline and conservative evaluation of sleep stages relying on strict criteria. Scoring was performed here by two trained neurophysiologists blind to experimental conditions who additionally verified that participants remained asleep after stimuli onset by tracking any

*Correspondence: sid.kouider@ens.fr
This is an open access article under the CC BY license (http://creativecommons.org/licenses/by/3.0/).

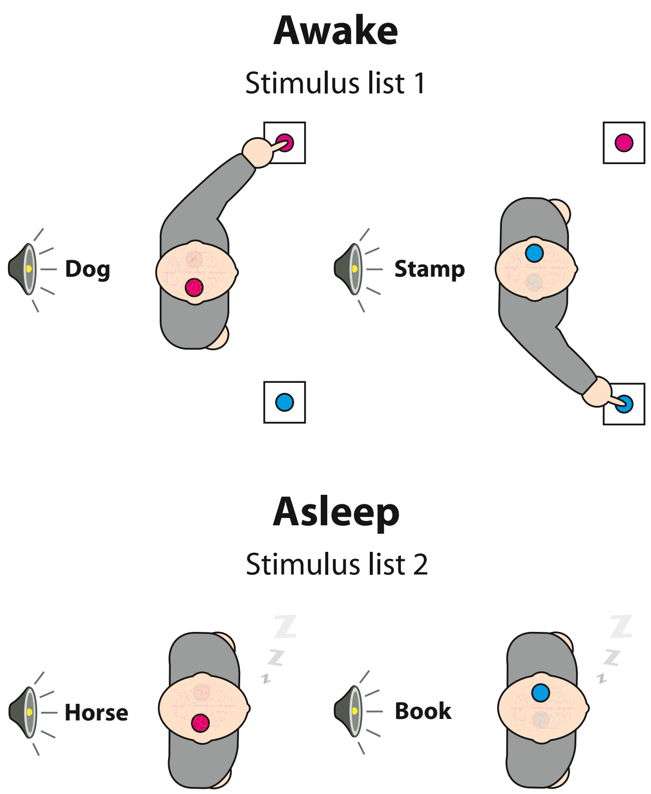

Figure 1. Schematic Description of the Induction Procedure

(A) Participants (n = 18 in each experiment) first made overt manual responses either to animal versus object names presented every 6 to 9 s (semantic decision task; experiment 1), or to words versus pseudowords (lexical decision task; experiment 2) while wearing an EEG cap.

(B) After participants either fell asleep (experiment 1) or entered the N2 stage (experiment 2), as assessed both by the absence of behavioral responses and by electrophysiological markers of sleep, a second list of stimuli was presented, and EEG indices of response preparation were used to evaluate covert classification.

See also Figures S1 and S4.

electrophysiological signs of arousals (reappearance of a wake-like EEG activity for more than 3 s [13], whether the trial was associated with a button press), or any microarousals. Details and statistics about sleep scoring are provided in the Supplemental Experimental Procedures (see also Figure S1 for individual sleep architectures).

LRPs constitute a direct and sensitive measure of response selection and preparation toward the target side, which is maximal in amplitude at scalp sites over the motor/premotor cortices contralateral to the responding hand [14, 15]. LRPs, traditionally computed by reference to response onset, can also be measured by reference to stimulus onset [16, 17], making them suitable to measure cortical responses in the absence of overt motor responses (i.e., during sleep). We first characterized the main (i.e., state-independent) effect of response preparation by collapsing sleep and wake trials and computing stimulus-locked LRPs using cluster-based permutation analysis (see the Supplemental Experimental Procedures). This analysis revealed a first negative deflection corresponding to the LRP, with two significant peaks at 660 and 1,620 ms, primarily over central (C3/C4) and central posterior (CP3/CP4) electrodes (Figure 2A). Interestingly, after 2,000 ms, the LRP returned to baseline for several seconds until the emergence of a second negative deflection peaking around 5,570 ms. Second, to test the difference between wake and sleep states, we subtracted the wake condition from the sleep condition (Figure 2B). Remarkably, we found no significant difference for the first LRP deflection but a clear significant effect afterward, during the opposite deflection, around 2,920 ms for C3/C4 and 3,800 ms for CP3/CP4. Restricted analysis for each vigilance state confirmed the significant early LRP deflection for wake trials and, crucially, also for sleep trials separately (Figures 2C and 2D). However, the opposite and later positive deflection was present only during wake trials. As shown in Figure 2C, the distribution of response times during wake trials suggests that the initial LRP reflects the preparation of the motor plan, while the inversion of potential appears to follow manual responses. This interpretation was confirmed by performance of a similar analysis on readiness potentials now time locked to the actual response showing the classical LRP deflection at response onset, followed immediately by the opposite deflection after the manual response (Figure S2). As discussed below, this opposite deflection in the wake condition is likely to reflect a postresponse checking mechanism that is exacerbated under conditions of drowsiness.

These results suggest that task-relevant motor preparation can be triggered during sleep. Yet, several potential issues should be addressed before drawing this conclusion. First, one might question whether participants in our study were truly asleep. Although our procedure for assessing sleep involved both online scoring and waiting for at least 2 min of absent responses before shifting to the new list of words, subjects sometimes pressed buttons either spontaneously or in response to auditory stimulation during the sleep list (14% of trials, not included in the analyses). Those button presses could be regarded as temporary arousals whereby the subjects might wake up for one or two trials, or even micro-arousals (i.e., less than 3 s). However, they might also reflect a nonconscious triggering of motor actions in responses to a sensory stimulation, as it is well-known in the literatures on visual masking (e.g., subliminal action priming [18]) and blindsight patients [19, 20]. In addition, past studies have shown that motor reflexes can be triggered during sleep [21]. Finally, these button presses might reflect, more simply, the fact that subjects during early sleep stages are prone to perform small movements considered in the literature as peripheral motor activations (i.e., unrelated to task or environmental contexts), such as muscle twitches [22]. Inspection of the data revealed that in most cases button presses were associated with micro-arousals, although there were cases where button presses were not accompanied by any signs of arousal. Importantly, we computed our sleep LRPs not only by excluding any trial with button presses, but also after performing a conservative evaluation of their vigilance state. Indeed, in order to be fully confident that the trials that we included in our analysis genuinely reflect a state of sleep, microarousals and arousals (associated with button presses or not) were detected and trials in the direct vicinity of these events were discarded, although they may be considered as sleep trials according to established guidelines. A related issue concerns the fact that our participants received the sleep list from the onset of NREM1, and thus our sleep condition reflects a mixture of NREM1 and NREM2 stages. Yet, contrary to NREM2, the NREM1 stage is sometimes regarded as an ambiguous transitory state in which awareness and responsiveness might be partially preserved [1, 7, 23]. Our data set did not allow us to reliably separate the two sleep stages due to a lack of power, as the 48

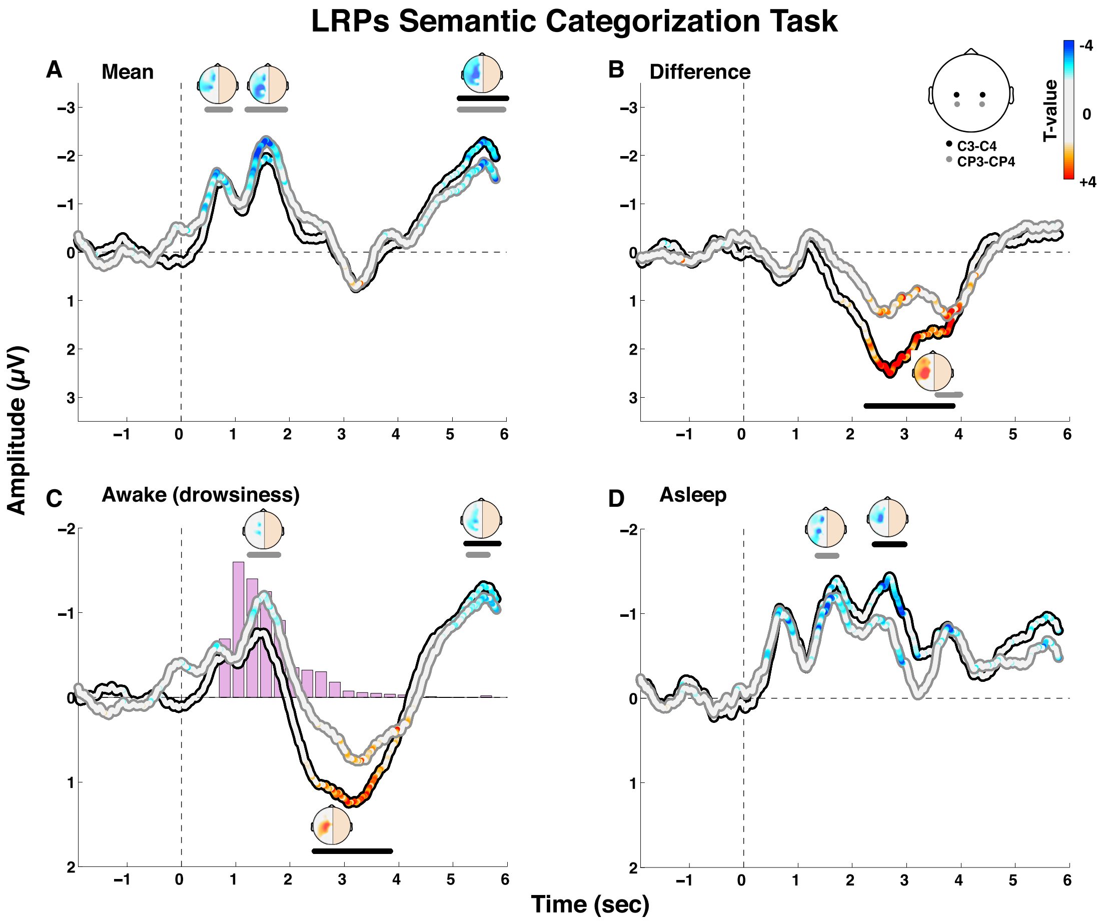

Figure 2. Semantic Categorization LRPs

LRPs, computed by subtraction of contralateral from ipsilateral activations (see the Supplemental Experimental Procedures), revealed covert response preparation toward the target side (i.e., contralateral to the appropriate hand movement) in the vicinity of motor areas, both during wake and sleep trials. Time series show the LRP curves from stimulus onset on central (C3/C4) and central posterior (CP3/CP4) electrodes for the main effect (A), the difference between sleep and wake conditions (B), and the LRPs restricted to each condition (C and D). Bars above the time series show significant clusters with a Monte Carlo p value <0.05. 2D topographies show the LRP over the whole scalp obtained for each couple of electrodes (i.e., left/right couples) during the peak of activation of each cluster (when both electrodes pairs reached significance at the same time, the topographies were identical in both peaks, and only one is shown for brevity). The color code shows significance at the sample level (time series) and electrode level (topographies), with white color on all nonsignificant data points ($p > 0.05$). Histograms in the wake LRP show the RTs distribution. See also Figures S2 and S3.

items were distributed across both stages. It thus remains possible that, even controlling for electrophysiological and behavioral markers of arousal, participants may have somehow remained conscious of the stimuli during trials scored as NREM1. To account for this potential issue and ensure that task-relevant responses can genuinely be triggered during sleep, we implemented a more stringent control of vigilance in the second experiment, where only NREM2 brain activity was considered in the sleep condition.

Another potential issue concerns the use in our study of a specific scoring method developed by Hori and collaborators for protocols with short epochs and focusing on hypnagogia [24, 25]. One might argue that this method, which is less commonly used, might underestimate the level of sleepiness and/or miss potential contaminations by microarousals in comparison to the standard scoring approach. We thus rescored our semantic decision data using the widely used guidelines of the American Academy of Sleep Medicine (AASM) [13]. We observed that the two scoring methods largely matched in terms of classifying trials in the wake or sleep state (93.1% overlap across participants; SD = 4.1%). Crucially, reanalyses of our data using the AASM scoring revealed a very similar pattern with a significant LRP deflection for sleep trials (see the Supplemental Experimental Procedures and the results in Figure S3), confirming the presence of task-relevant responses during sleep even when a more conventional method for scoring sleep was used. Finally, regarding the comparison with the wake state, a potential issue might be that the strong positive deflection that we observed with a reversal of the LRP response after an overt motor response might reflect specific conditions of drowsiness. Indeed, participants were tested while falling asleep and reaching a certain level of drowsiness, which might increase the reliance on postresponse checking mechanisms, leading to the reconfiguration and amplification/reduction of ipsi-/contralateral motor areas [26, 27]. Hence, a more direct comparison between wake and sleep states would thus not only exclude NREM1 trials as described above, but also compare sleep with conditions of full wakefulness (i.e., avoiding the drowsiness period where subjects are in the process of falling asleep).

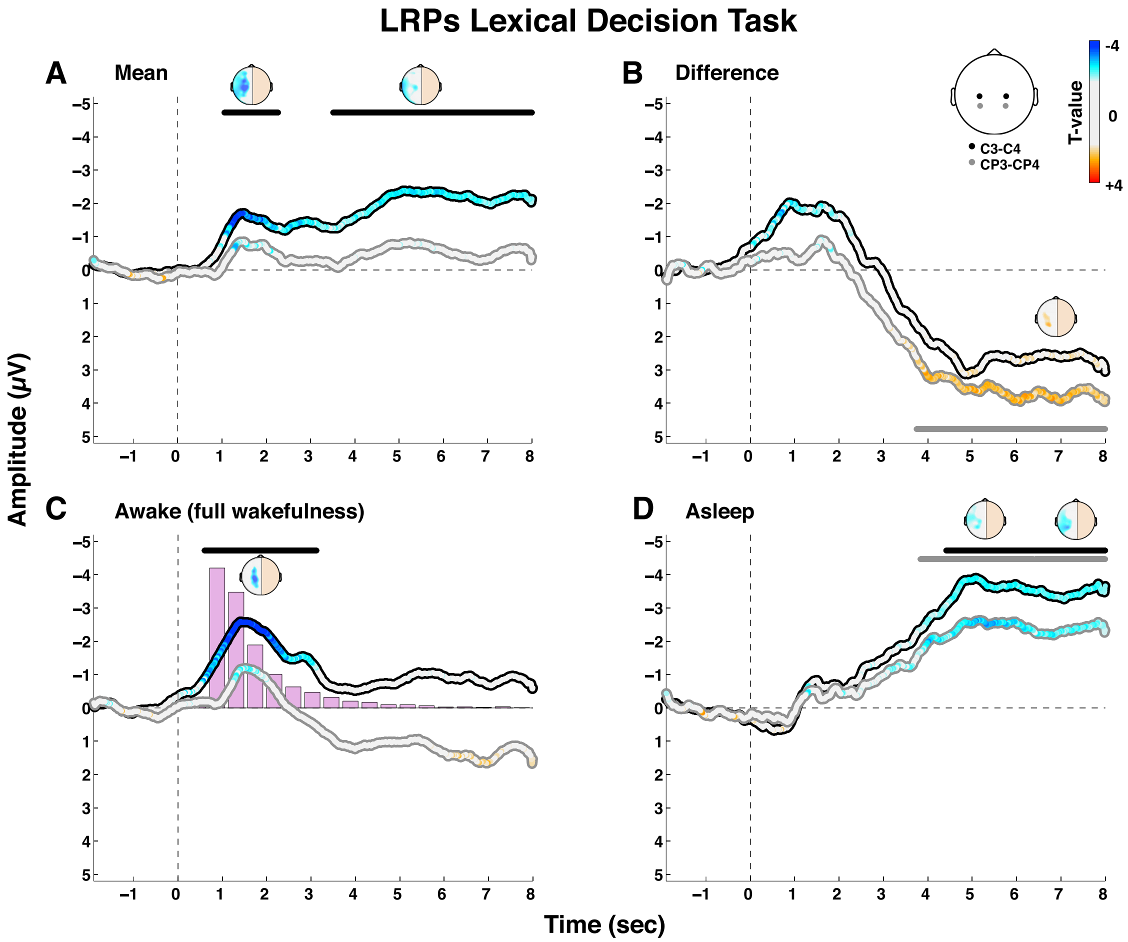

Figure 3. Lexical Decision LRPs

See Figure 2 for a description of (A)–(D). See also Figure S2.

LRPs for Lexical Decision in Full Wakefulness and NREM2 Sleep

We performed a second experiment in which we instructed participants to perform a lexical decision on spoken material. Participants classified auditory stimuli as words versus pseudowords (i.e., items that don't exist in the lexicon but share the same phonological properties as real words) with their left versus right hand (counterbalanced across subjects). This second experiment, in addition to dealing with the potential issues mentioned above regarding the wake-sleep transition, allowed us to verify whether the induction approach can be generalized to other classification tasks on external stimuli. Here, the nap was preceded by a session in which participants received the first list of stimuli under full wakefulness while sitting upright and not being allowed to fall asleep. Participants were then presented repeatedly with the same list while being reclined and allowed to fall asleep under similar testing conditions as in the semantic decision group. In addition, participants in this second experiment received the second list of stimuli (n = 72) only after the onset of the NREM2 stage (i.e., after the first appearance of a spontaneous K complex or sleep spindle). This design allowed contrasting LRPs during consolidated sleep versus full wakefulness rather than during the transition, where subjects are either drowsy or in a labile (NREM1) sleep stage. Since the Hori scoring is optimized for evaluating the hypnagogic period (primarily NREM1), we decided to apply AASM scoring for this second experiment while also controlling for microarousals (see the Supplemental Experimental Procedures and Figure S4 for individual hypnograms). Trials associated with a button press and microarousals dropped to 2.3% (SD = 1.7%) and 0.3% (SD = 0.6%), respectively, and were excluded from further analysis.

Analysis of the main (i.e., state-independent) effect of response preparation revealed two LRP clusters, with an early effect peaking at 1,276 ms and a later and more sustained effect peaking at 5,016 ms and extending from 3,508 ms until the end of the epoch at 8,000 ms (Figure 3A). Separate analyses for each vigilance state showed that the early LRP component was mostly driven by the wake condition, whereas the later and more sustained cluster was primarily driven by the sleep condition (Figures 3C and 3D). Indeed, whereas the LRP in the wake condition was rather transient and overlapped with the reaction times distribution, the LRP during sleep corresponded to a large and sustained response developing slowly over time. As a consequence, the contrast of wakefulness (i.e., the difference between wake and sleep trials; Figure 3B) revealed a trend for an early negativity, suggesting a stronger early LRP under wake conditions, and a significant and sustained positivity for the late component, reflecting a delayed LRP during sleep. These results confirm the presence of covert task-relevant responses to speech during sleep and extend the finding in the semantic

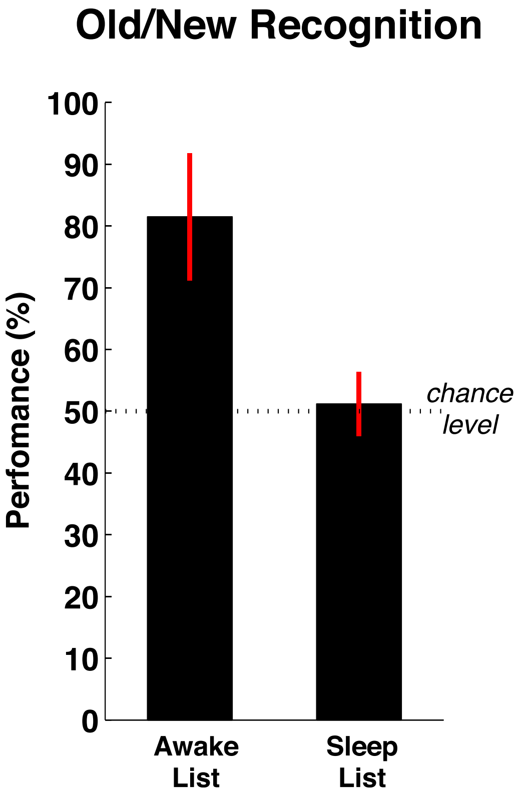

Figure 4. Results of the Old/New Explicit Recognition Test Performed Immediately after the Nap

Participants received stimuli from the wake list, sleep list, and an entirely new list and were instructed to indicate which items had been played previously or were entirely new. Performance, computed by comparison of responses of the wake and sleep lists to the new list, revealed high-accuracy performance for the wake list but chance-level performance for stimuli presented during sleep. Error bars indicate 1 SD.

decision from experiment 1 to the classification of lexical properties during the NREM2 state. Notably, the opposite deflection found in experiment 1 under drowsy conditions was not observed here under conditions of full wakefulness. It is also interesting to observe that the LRP during sleep was further delayed in time compared to experiment 1. We interpreted this finding as the involvement of slower mechanisms of evidence accumulation during the N2 stages in experiment 2, compared to the mixture of N1 and N2 responses in experiment 1 [1].

One might still argue that participants in our study were somewhat aware of the spoken stimuli, with fleeting microarousals that are difficult to detect in the EEG, resulting in a state of transient arousal/drowsiness not allowing them to perform an overt behavioral response. In order to directly address this issue, through an operational measure of stimulus awareness, we instructed participants to perform an explicit recognition task right after the lexical decision experiment, after regaining full consciousness. They were presented with the stimuli from the wake list, from the sleep list, or from a new list of completely novel items (counterbalanced across participants) and were instructed to classify each stimulus as either old or new and then rate their confidence about their decision on a scale ranging from 1 (completely guessing) to 7 (completely sure). Results of the posttest (see Figure 4) revealed that participants could distinguish new words from words presented during the wake period (performance = 81.5%, $d' = 2.16$, both $p < 0.0001$) but, crucially, not from words presented during sleep (performance = 51.2%, $d' = 0.13$, both nonsignificant [n.s.]). Consistently, the postdecision confidence estimates also did not differ between the new and sleep lists (mean confidence = 4.79 versus 4.80, respectively; n.s.), whereas they were significantly higher for the list presented during the preceding period of wakefulness (5.80, both $p < 0.001$). Overall, these results add strong evidence supporting the fact that participants did not have explicit access to the stimuli presented during sleep and confirm that the LRP obtained during sleep most likely reflects a nonconscious form of speech processing.

Discussion

There is now converging evidence that environmental stimuli can still be processed during sleep, at least to a certain degree [2]. For instance, meaningful stimuli (e.g., own names, own baby's cry, and fire alarm) are more likely to lead to awakening [28–31]. Furthermore, sleeping participants, while in REM or NREM stages, can create novel sensory associations between tones and odors [3] or reactivate existing semantic associations as evidenced by the presence of an N400 component in EEG [4–7]. Besides, sleepwalkers are able to re-enact recently learned sequences of movements [32]. Thus, there is evidence, albeit scarce, that sleep does not preclude meaning extraction or the activation of learned associations and sensorimotor mappings. However, to date, no study has directly tested the possibility that environmental stimuli are processed in a flexible manner, all the way up to the preparation of task-relevant responses. Here, using LRPs, we show that sleeping participants are still able to prepare for the appropriate response on semantic and lexical decision tasks practiced before falling asleep. The current design, using single-word presentations, does not directly test for meaning extraction (unlike classical N400 paradigms using word pairs or sentences). However, our study reveals speech processing through semantic and lexical categorization by demonstrating the preparation of motor plans conditional on the meaning of spoken words. These results not only confirm previous findings showing that semantic information can still be extracted during sleep, but further show that this nonconscious meaning extraction can be routed by the task context and reach higher processing levels, up to motor preparation stages. This suggests that when processing environmental information during sleep, at least during early NREM stages, only the final stages related to action execution might be suppressed.

An important remaining question, therefore, is where in the neural stream ranging from motor preparation to action execution lays the bottleneck responsible for the lack of behavioral responses. Previous studies revealed that sleep is associated with both the inhibition of dorsolateral prefrontal cortex, a crucial area for executive functions [8, 9], and the functional breakdown in thalamocortical connectivity, associated with the loss of wakefulness and sensory awareness [33]. On the contrary, neural activity in other cortical regions, including sensorimotor areas, does not importantly differ from the wake stage [9, 34, 35]. The preserved functionality of these regions may support elaborate—albeit automatized—cognitive processes such as those observed in the present study. One might even expect that, as long as a given task has been induced during the wake stage, almost any processing stream

could potentially remain activated during sleep. Future studies will be necessary to address this issue and, in particular, whether even higher-order regions dealing with executive functions such as cognitive control or task switching can be triggered using a task-induction strategy.

It remains to be elucidated whether this finding would generalize to other sleep stages, and in particular to REM sleep, in which there is an almost complete muscular paralysis but electrophysiological activity is closer to that of wakefulness. On the one side, because the strong inhibition of motor neurons during REM sleep involves only subcortical structures (such as the locus coeruleus, which targets motor neurons in the spinal cord), and given the relatively preserved information processing capabilities during this stage [36], one might still expect similar covert responses as found here. On the other side, these findings might be restricted to the initial stages of sleep, during which the thalamus is mostly deactivated whereas large parts of the cortex remain active [37]. Future studies relying on full-night protocols will be necessary to address whether the integration of semantic and decision processes can bypass early sleep stages.

Beyond revealing unsuspected processing capabilities in the sleeping brain, this study uncovers a promising avenue to study nonconscious processes. Research investigating the distinction between conscious and nonconscious mechanisms (the so-called "contrastive approach" [38]) generally focuses on the notion of contents of consciousness. In this framework, the participant can be nonconscious "of" a specific content as in a typical situation of visual masking but remains fully conscious in the intransitive sense of being aroused and vigilant. For instance, although previous studies using subliminal priming have shown that invisible primes can trigger lateralized readiness potentials [10, 39], participants in these studies were still having conscious access to their goal-directed behaviors in order to perform a specific task on target stimuli. Here, although sleeping participants may continue to process information in a goal-oriented manner, this task set is presumably maintained without the participant being conscious of it. Moreover, our experimental approach relying on levels rather than contents of consciousness not only allows examination of the neural consequences of perceptual processes when the subject is nonconscious in any possible respects, but also offers the opportunity to use sensory stimuli that are not degraded in any manner. Indeed, the strong degradation of sensory signals typically used to achieve robust unawareness in masking studies, either in the visual [11] or the auditory modality [40], unavoidably decreases the strength of neural responses, especially in brain regions dealing with high-level information [11]. Hence, studying sleep in this context allows pushing further the limits and extents of nonconscious processes and establishing the properties of a broader and more natural type of cognitive unconscious.

Supplemental Information

Supplemental Information includes Supplemental Experimental Procedures and four figures and can be found with this article online at http://dx.doi.org/10.1016/j.cub.2014.08.016.

Acknowledgments

This research was supported by ANR grants to S.K. and to the Institute of Cognitive Studies (ANR-10-BLAN-1902, ANR-10-LABX-0087, and ANR-10-IDEX-0001-02), a grant from the Ministère de l'Enseignement Supérieure et de la Recherche to T.A., and a Wellcome Trust biomedical research fellowship (WT093811MA) to T.A.B. We thank Karalyn Patterson and Rachel Holland for assisting with an early version of the stimuli in experiment 1.

Received: February 14, 2013
Revised: July 7, 2014
Accepted: August 8, 2014
Published: September 11, 2014

References

1. Ogilvie, R.D. (2001). The process of falling asleep. Sleep Med. Rev. *5*, 247–270.
2. Hennevin, E., Huetz, C., and Edeline, J.M. (2007). Neural representations during sleep: from sensory processing to memory traces. Neurobiol. Learn. Mem. *87*, 416–440.
3. Arzi, A., Shedlesky, L., Ben-Shaul, M., Nasser, K., Oksenberg, A., Hairston, I.S., and Sobel, N. (2012). Humans can learn new information during sleep. Nat. Neurosci. *15*, 1460–1465.
4. Brualla, J., Romero, M.F., Serrano, M., and Valdizán, J.R. (1998). Auditory event-related potentials to semantic priming during sleep. Electroencephalogr. Clin. Neurophysiol. *108*, 283–290.
5. Bastuji, H., Perrin, F., and Garcia-Larrea, L. (2002). Semantic analysis of auditory input during sleep: studies with event related potentials. Int. J. Psychophysiol. *46*, 243–255.
6. Ibáñez, A., López, V., and Cornejo, C. (2006). ERPs and contextual semantic discrimination: degrees of congruence in wakefulness and sleep. Brain Lang. *98*, 264–275.
7. Goupil, L., and Bekinschtein, T.A. (2012). Cognitive processing during the transition to sleep. Arch. Ital. Biol. *150*, 140–154.
8. Muzur, A., Pace-Schott, E.F., and Hobson, J.A. (2002). The prefrontal cortex in sleep. Trends Cogn. Sci. *6*, 475–481.
9. Maquet, P. (2000). Functional neuroimaging of normal human sleep by positron emission tomography. J. Sleep Res. *9*, 207–231.
10. Dehaene, S., Naccache, L., Le Clec'H, G., Koechlin, E., Mueller, M., Dehaene-Lambertz, G., van de Moortele, P.F., and Le Bihan, D. (1998). Imaging unconscious semantic priming. Nature *395*, 597–600.
11. Kouider, S., and Dehaene, S. (2007). Levels of processing during non-conscious perception: a critical review of visual masking. Philos. Trans. R. Soc. Lond. B Biol. Sci. *362*, 857–875.
12. Masaki, H., Wild-Wall, N., Sangals, J., and Sommer, W. (2004). The functional locus of the lateralized readiness potential. Psychophysiology *41*, 220–230.
13. Iber, C., Ancoli-Israel, S., Chesson, A., and Quan, S.F. (2007). The AASM Manual for the Scoring of Sleep and Associated Events: Rules, Terminology and Technical Specifications (Westchester: American Academy of Sleep Medicine).
14. Kutas, M., and Donchin, E. (1980). Preparation to respond as manifested by movement-related brain potentials. Brain Res. *202*, 95–115.
15. Coles, M.G. (1989). Modern mind-brain reading: psychophysiology, physiology, and cognition. Psychophysiology *26*, 251–269.
16. Leuthold, H. (2003). Programming of expected and unexpected movements: effects on the onset of the lateralized readiness potential. Acta Psychol. (Amst.) *114*, 83–100.
17. Töllner, T., Rangelov, D., and Müller, H.J. (2012). How the speed of motor-response decisions, but not focal-attentional selection, differs as a function of task set and target prevalence. Proc. Natl. Acad. Sci. USA *109*, E1990–E1999.
18. Vorberg, D., Mattler, U., Heinecke, A., Schmidt, T., and Schwarzbach, J. (2003). Different time courses for visual perception and action priming. Proc. Natl. Acad. Sci. USA *100*, 6275–6280.
19. Weiskrantz, L. (1986). Blindsight: A Case Study and Implications (Oxford: Clarendon Press).
20. Cowey, A., and Stoerig, P. (2004). Stimulus cueing in blindsight. Prog. Brain Res. *144*, 261–277.
21. Dagnino, N., Loeb, C., Massazza, G., and Sacco, G. (1969). Hypnic physiological myoclonias in man: an EEG-EMG study in normals and neurological patients. Eur. Neurol. *2*, 47–58.
22. Chase, M.H., and Morales, F.R. (2005). Control of motoneurons during sleep. In Principles and Practice of Sleep Medicine, M.K. Kryger, T. Roth, and W.C. Dement, eds. (New York: Saunders), pp. 154–168.
23. Bareham, C.A., Manly, T., Pustovaya, O.V., Scott, S.K., and Bekinschtein, T.A. (2014). Losing the left side of the world: rightward shift in human spatial attention with sleep onset. Sci Rep *4*, 5092.

24. Hori, T., Hayashi, M., and Morikawa, T. (1994). Topographical EEG changes and the hypnagogic experience. In Sleep Onset: Normal and Abnormal Processes, R.B. Ogilvie and J. harsh, eds. (Washington: American Psychological Association), pp. 237–253.
25. Tanaka, H., Hayashi, M., and Hori, T. (1997). Topographical characteristics and principal component structure of the hypnagogic EEG. Sleep *20*, 523–534.
26. Praamstra, P., Boutsen, L., and Humphreys, G.W. (2005). Frontoparietal control of spatial attention and motor intention in human EEG. J. Neurophysiol. *94*, 764–774.
27. Haggard, P. (2005). Conscious intention and motor cognition. Trends Cogn. Sci. *9*, 290–295.
28. Oswald, I., Taylor, A.M., and Treisman, M. (1960). Discriminative responses to stimulation during human sleep. Brain *83*, 440–453.
29. Formby, D. (1967). Maternal recognition of infant's cry. Dev. Med. Child Neurol. *9*, 293–298.
30. Bruck, D., Ball, M., Thomas, I., and Rouillard, V. (2009). How does the pitch and pattern of a signal affect auditory arousal thresholds? J. Sleep Res. *18*, 196–203.
31. Portas, C.M., Krakow, K., Allen, P., Josephs, O., Armony, J.L., and Frith, C.D. (2000). Auditory processing across the sleep-wake cycle: simultaneous EEG and fMRI monitoring in humans. Neuron *28*, 991–999.
32. Oudiette, D., Constantinescu, I., Leclair-Visonneau, L., Vidailhet, M., Schwartz, S., and Arnulf, I. (2011). Evidence for the re-enactment of a recently learned behavior during sleepwalking. PLoS ONE *6*, e18056.
33. Massimini, M., Ferrarelli, F., Huber, R., Esser, S.K., Singh, H., and Tononi, G. (2005). Breakdown of cortical effective connectivity during sleep. Science *309*, 2228–2232.
34. Issa, E.B., and Wang, X. (2008). Sensory responses during sleep in primate primary and secondary auditory cortex. J. Neurosci. *28*, 14467–14480.
35. Nir, Y., Vyazovskiy, V.V., Cirelli, C., Banks, M.I., and Tononi, G. (2013). Auditory responses and stimulus-specific adaptation in rat auditory cortex are preserved across NREM and REM sleep. Cereb. Cortex. Published online December 8, 2013. http://dx.doi.org/10.1093/cercor/bht328.
36. Chennu, S., and Bekinschtein, T.A. (2012). Arousal modulates auditory attention and awareness: insights from sleep, sedation, and disorders of consciousness. Front. Psychol. *3*, 65.
37. Magnin, M., Rey, M., Bastuji, H., Guillemant, P., Mauguière, F., and Garcia-Larrea, L. (2010). Thalamic deactivation at sleep onset precedes that of the cerebral cortex in humans. Proc. Natl. Acad. Sci. USA *107*, 3829–3833.
38. Baars, B.J. (1989). A Cognitive Theory of Consciousness (Cambridge: Cambridge University Press).
39. Eimer, M., and Schlaghecken, F. (1998). Effects of masked stimuli on motor activation: behavioral and electrophysiological evidence. J. Exp. Psychol. Hum. Percept. Perform. *24*, 1737–1747.
40. Kouider, S., and Dupoux, E. (2005). Subliminal speech priming. Psychol. Sci. *16*, 617–625.

Current Biology, Volume *24*

Supplemental Information

Inducing Task-Relevant Responses
to Speech in the Sleeping Brain

Sid Kouider, Thomas Andrillon, Leonardo S. Barbosa, Louise Goupil, and Tristan A. Bekinschtein

Supplemental data

Figure S1. ***Top*****. Average power spectra across participants for wake trials (blue) and sleep trials (red) in Experiment 1.** Note the turnaround between alpha (8-13 Hz) and beta (20-40 Hz) rhythms predominant in wakefulness and sleep-related oscillations (delta (0.1-4 Hz), theta (4-7 Hz), spindle range (11-16 Hz)). Power spectra were computed on C3/C4 referenced to the mastoids with a fast Fourier transform and averaged across subjects. *Purple bars* mark frequencies for which power was significantly higher in sleep compared to wake (paired t-test, 0.05, false detection rate correction). *Light green bars* mark frequencies for which power was significantly higher in wake compared to sleep (paired t-test, 0.05, false detection rate correction). Red and blue shadings denote standard-error to the mean. ***Bottom*****.** **Individual hypnograms.** *Black lines* show the vigilance state per trial visually scored using AASM guidelines. *Grey dots* show recorded response times (RTs). Note the large variability in RTs typical of drowsiness. The line below each hypnogram contains information about the stimulus list (*cyan*: wake list; *magenta*: sleep list) and the line above each hypnogram depicts the final scoring taking into account behavioral and electrophysiological criteria (*blue*: “wake” trials ; *red*: “sleep” trials). Related to Figure 1.

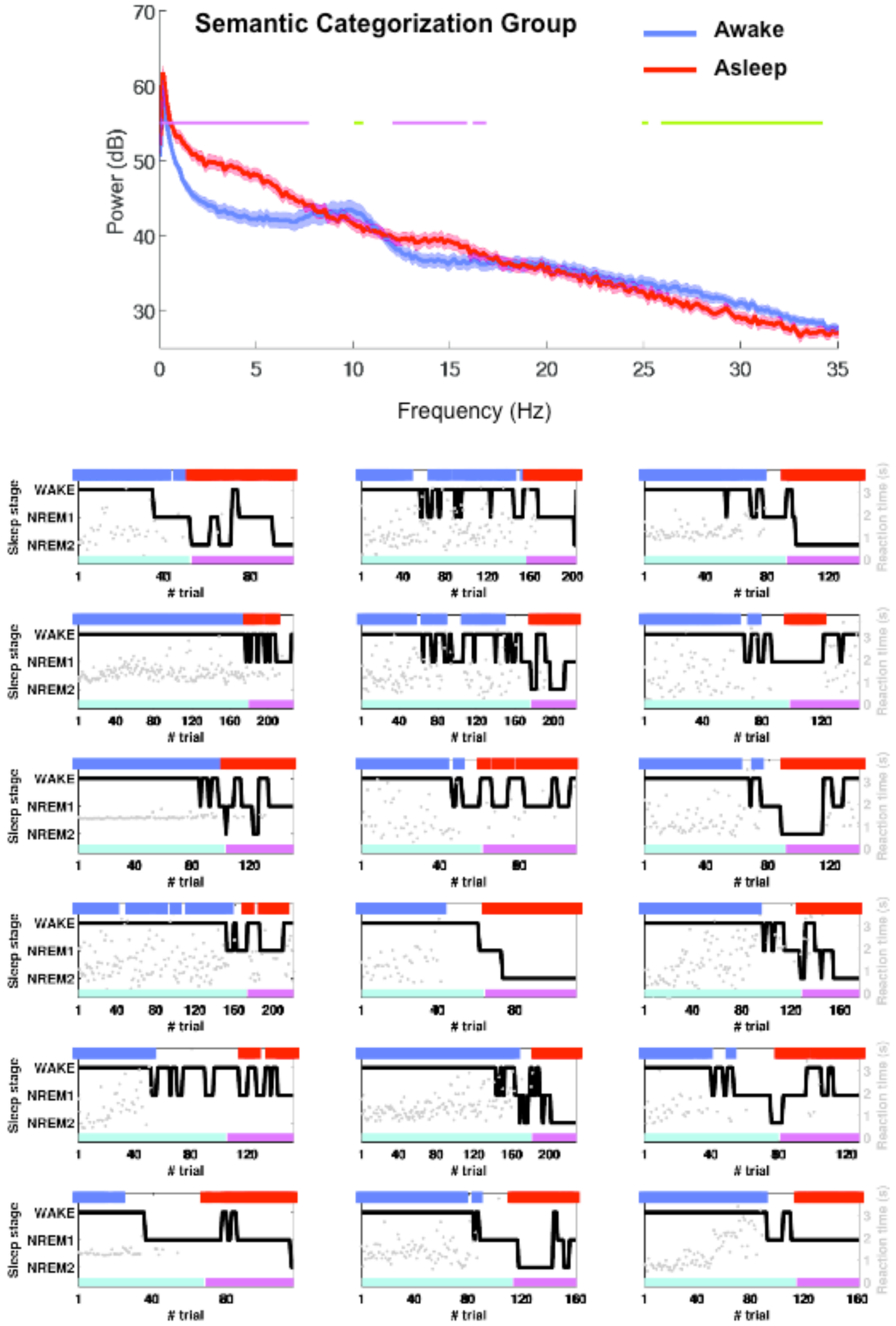

Semantic Categorization Group
Awake
Asleep
Power (dB)
Frequency (Hz)
Sleep stage
WAKE
NREM1
NREM2
trial
Reaction time (s)

Figure S2. Response-locked LRPs for Experiment 1 (top panel) and Experiment 2 (bottom panel). LRPs were here averaged with respect to the participant response on each trial (i.e. 0 ms corresponds to the response time). Baseline correction was performed with respect to a -4000 to -2000ms period before stimulus onset. Time-series show the LRP curves on central (C3/C4) and central posterior (CP3/CP4) electrodes (See Figure 2 for more details). Related to Figure 2 and Figure 3.

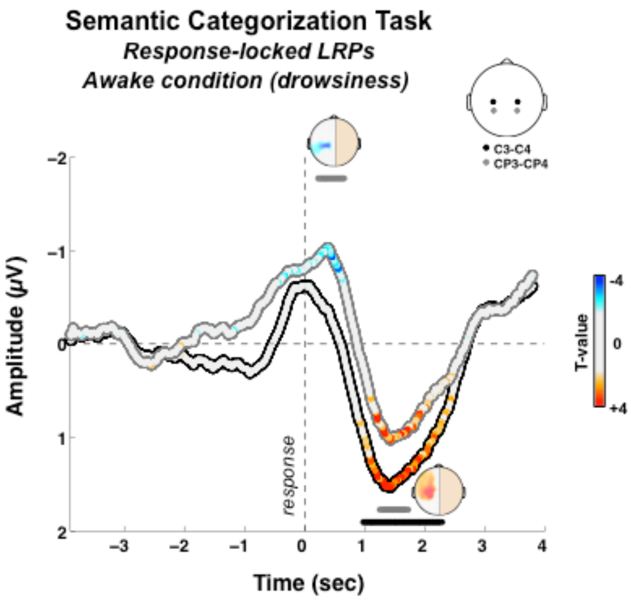

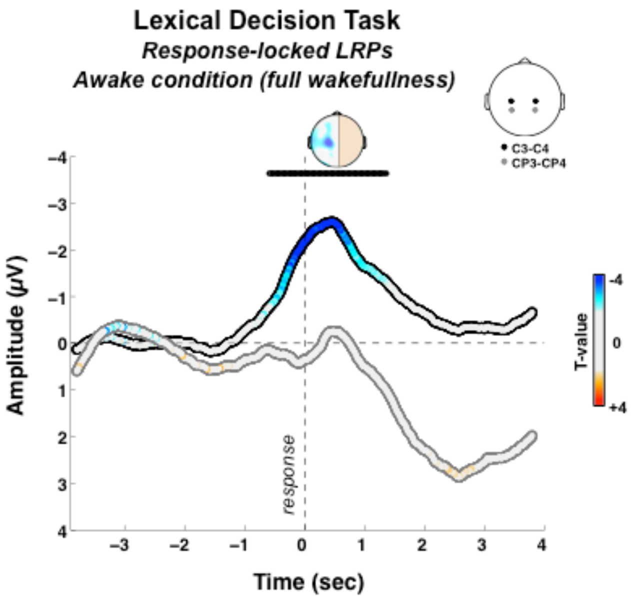

Figure S3. Stimulus-locked LRPs in Experiment 1 with standard sleep scoring. LRPs for the sleep condition in which trials were scored according to standard guidelines (see section on "Supplemental sleep scoring using standard guidelines" and Figure 2 for more details). Related to Figure 2.

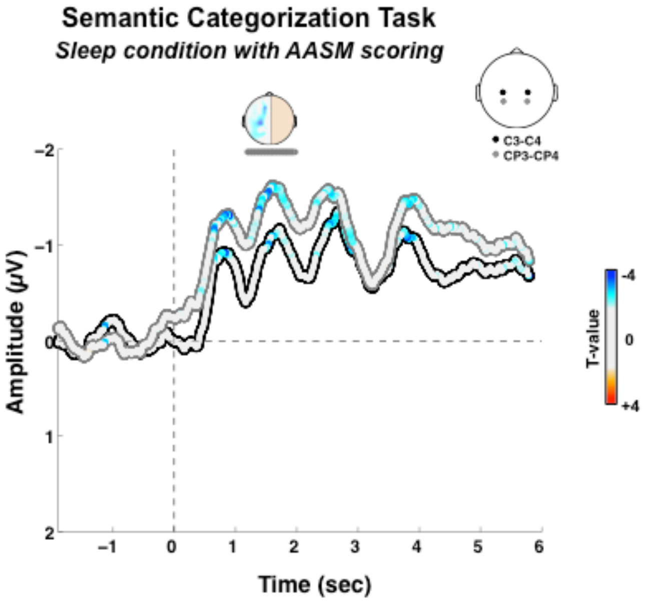

Figure S4. *Top.* Average power spectra across participants for wake trials (blue) and sleep trials (red) in Experiment 2. *Bottom*. Individual hypnograms See Figure S1 for details. Related to Figure 1.

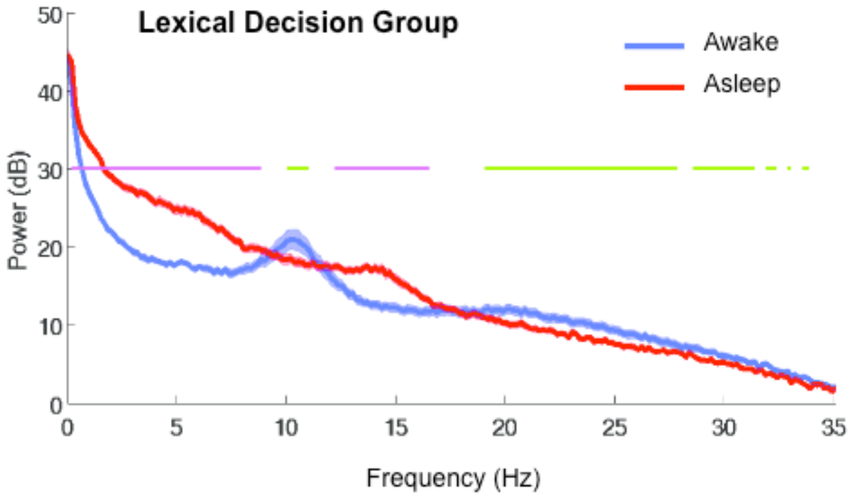

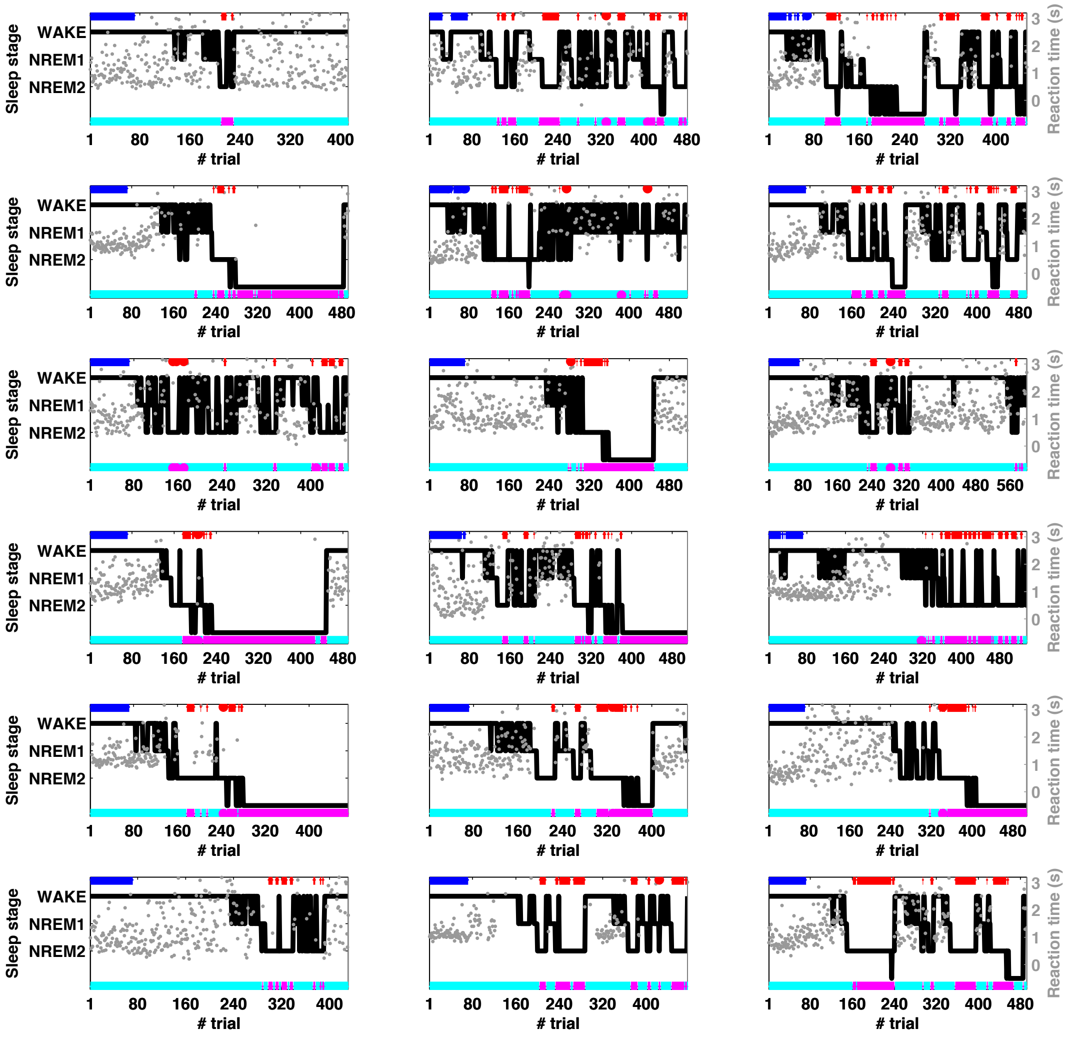

Sleep stage
WAKE
NREM1
NREM2
trial
Reaction time (s)

Supplemental Experimental Procedures

Participants. Eighteen native English speakers (6 women and 12 men, age range: 18-30 years) took part in Experiment 1. An additional 29 participants were tested but not included in the final analysis because of a failure to fall asleep (N=27) or due to excessive artefacts in the EEG signal (N=2). For Experiment 2, 18 native French speakers (12 women and 4 men, age range: 20-28 years) were included out of 22 subjects. Four subjects were thus excluded either due to not falling asleep (N=1) or note reaching the N2 stages (N=3). All subjects were right-handed, and reported no auditory, neurological or psychiatric alterations. To increase the probability that participants would fall asleep in our experimental setup, only easy sleepers, as assessed by the Epworth Sleepiness Scale, were selected for this study. This scale evaluates whether participants are used to easily falling asleep, for instance when watching TV or during train trips. Recruited participants were considered healthy with relatively high ESS scores but not corresponding to a condition of pathological sleep such as hypersomnia :the average ESS scores were 10.4 with (range 7-14) for Experiment 1, and 11.6 (range 7-16) for Experiment 2, while the maximum possible score is 24. Participants were also asked to avoid exciting substances as coffee, and to sleep 1-2 hours (20%) less than usual the night preceding experiment 1 and 2-3 hours (30%) less than usual for Experiment 2. They signed a written consent and were paid for their participation. Both experiments were approved by the relevant local ethical committees (Cambridge psychology research ethics committee for Experiment 1, Conseil d'évaluation éthique pour les recherches en santé for Experiment 2).

Stimuli. For Experiment 1, stimuli were spoken words selected from the CELEX lexical database (Linguistic Data Consortium, University of Pennsylvania). There were 48 names of objects and 48 names of animals. Half were monosyllabic and the other half disyllabic, with animal and object names matched as closely as possible in terms of combined (spoken and written) log lemma frequencies, as confirmed by an independent t-test ($p > 0.10$). Additionally, words within the two categories were matched in a pair-wise fashion regarding their phonological properties: each object name was matched with a similar animal name (for example “quilt” was matched with “quail”),

ensuring that animal and objects names could not be differentiated in terms of sub-semantic (i.e., phonological) properties. The words were tape-recorded by a female voice and digitized. Two lists of 48 stimuli each were produced, one for the wake period and the other for the sleeping period (counterbalanced across participants). For Experiment 2, the material consisted of 216 auditory stimuli corresponding to 108 pairs of words and pseudowords (half CVC monosyllabic and half CV-CV disyllabic) recorded by a male native French speaker and digitized. Within each pair, words and pseudowords were matched in length and phonological (consonant-vowel) structure. The words were selected from the Lexique database [S1] and the pseudowords were all legal and pronounceable combinations of sounds in French. Three lists of 72 stimuli matched for frequency and phonological structure were constructed such as to be counterbalanced across participants for the wake period, the sleep period and the new list in the old/new recognition task following the main experiment.

Procedure. Participants were lying down with their eyes closed in a comfortable reclining chair in a dark and electrically and acoustically shielded EEG cabin. Stimuli were presented binaurally through headphones (Experiment 1) or through loud speakers (Experiment 2). Participants were instructed to perform a semantic categorization on whether each spoken word referred to an animal or to an object (Experiment 1) or to perform a lexical decision on whether each spoken stimulus existed or not in French (Experiment 2), by pressing a button with either their left or right hand (with response hand counterbalanced between participants). For Experiment 1, they were told that they could fall asleep at any time during the task, but were asked not to stop responding deliberately before falling asleep (i.e. not to stop responding in order to fall asleep). For Experiment 2, participants first performed a full session with the wake list items (about 10 minutes) under conditions were they were fully wake and not allowed to fall asleep, before hearing the wake list again while being reclined and allowed to fall asleep under similar testing conditions as in Experiment 1. Testing conditions encouraged the transition towards sleep while remaining engaged with the same task-set (explicit allowance to fall asleep, dark room, eyes closed, reclining chair, several repetitions of the first stimulus list, long inter stimulus interval). The continuous, uninterrupted flow within and across the two lists of stimuli was aimed at reducing the probability of awakening.

While being awake, participants could hear up to 4 repetitions of the first list. In Experiment 1, they were presented with the second list of 48 items only once during sleep while in Experiment 2, they could receive the second list of 72 items up to 3 times to increase the number of sleep trials. Stimuli were presented in a random order with an inter-stimulus interval varying between 6 and 9 seconds in Experiment 1 and a fixed duration of 9 seconds for Experiment 2. The presentation of spoken items would switch to the second list without interrupting the pace of the experiment whenever the participant was assessed by the experimenter as being asleep (Experiment 1) or as entering the NREM2 stage (Experiment 2, see details below), For Experiment 2, stimulation was switched back to the wake list in cases of return to NREM1, (micro)-awakenings and/or button presses. Stimulus delivery and response collection was controlled by the E-Prime software (Psychology Software Tools, Pittsburgh, PA) for Experiment 1 and by the Matlab (MathWorks Inc. Natick, MA, USA) using the Psychophysics Toolbox [S2] for Experiment 2.

EEG recordings and analysis. The electroencephalogram was continuously recorded from 64 Ag/AgCl electrodes (NeuroScan Labs system for Experiment 1; Electrical Gegodesic Inc system for Experiment 2), with Cz as a reference. The impedance for electrodes was kept following constructor recommendations. Data were acquired with a sampling rate of 500 Hz (Experiment 1) or 250 Hz (Experiment 2). For the wake trials, only the first list occurrence was analysed (Experiment 1: N=48, Experiment 2: N=72). Continuous data were epoched from -2000 to 6000ms (Experiment 1) or to 8000ms (Experiment 2) in relation to stimulus onset, low-pass filtered at 30Hz and baseline corrected in respect to the pre-stimulus window of 2000ms. Trials with any electrode passing an absolute threshold (Experiment 1 with the NeuroScan system: 1000 μV, Experiment 2 with the Electrical Gegodesic Inc system: 250 μV) were rejected from the analysis (this concerned only non-physiological events). We used a very liberal threshold because sleep trials may contain large-magnitude K-complexes.

Separate averages were computed for left (L) and right (R) hand trials, resulting in two average waveforms for each electrode and participant. Stimulus locked LRP were then computed according to the procedure by Coles (1989, [S3]), using the ERP waveforms recorded from corresponding electrode pairs in each hemisphere as follow:

LRP = [(R hand – L hand trials) on L electrode + (L hand – R hand trials) on R electrode] * 0.5

Statistical significance was assessed through cluster/permutation statistics calculated within participants, allowing us to deal with the potential issue of multiple comparisons in a principled manner. Each cluster was constituted by the samples that consecutively passed a specified threshold (in this case sample p-value of 0.1). As demonstrated by Maris & Oostenveld (2007, [S4]), this threshold doesn't change the type-1 error, and the method controls for false alarms independent of this value. The cluster statistics was chosen as the sum of the t-values of all the samples in the cluster. Then, we compared the cluster statistics of each cluster with the maximum cluster statistics of 1000 random permutations. The significance of LRPs was assessed during both for the wake and sleep conditions by using a threshold monte-carlo p-value of 0.05.

Sleep assessment for Experiment 1. Sleep onset was determined *online* by relying on both behavioural and electrophysiological criteria. Participants were assumed to be asleep if they were not responding for at least 2 minutes, and if they were presenting EEG and EOG patterns characteristic of NREM sleep: reduction of fast rhythms (alpha – beta) in favour of slower rhythms (theta waves), slow-eye movements, vertex sharp waves and possibly evoked and/or spontaneous K-complexes and sleep spindles. Once sleep onset was confirmed, the first list was switched to a second one, never heard by the participant. For Experiment 1, after switching list, participants could occasionally press a button (14% of the trials in the sleep list). An offline sleep assessment was therefore conducted to confirm the sleeping state and to remove arousals or ambiguous trials (i.e., with potential micro-arousals), as well as trials with a button press. For Experiment 1, in which we concentrated on wake-to-sleep transition, we used an extension of standard sleep staging adapted and validated by Hori and

collaborators [S5, S6]. This method allows for a more refined sleep scoring since it uses smaller epochs prior to the stimulus onset (4 seconds) and allows for a more detailed characterisation of the hypnagogic period at the time of the auditory stimulation. Wakefulness was characterized by regular responses to stimuli, presence of fast low-amplitude rhythms such as alpha rhythms (8-13 Hz) especially on occipital electrodes, eye-blinks or saccades. Participants were declared asleep after the disappearance of alpha rhythm, replaced by slower oscillations (vertex sharp waves, theta rhythms). On the EOG, presence of slow eye movements was also indicative of the wake to NREM1 transition. Finally, when spontaneous K-complexes or spindles occurred in the 4s epoch prior to stimulus onset, the trial was scored as NREM2.

Importantly, in our protocol, it was crucial to assess not only the context in which stimuli were played (determined through the careful examination of the pre-stimulus activity) but also how these stimuli affected brain activity by potentially triggering micro-arousals. In order to retain as sleep trials only those for which participants were genuinely asleep and remained in this state, we visually detected and marked every sign of arousal (increase in low-amplitude fast rhythms such as alpha oscillations or oscillations above 16Hz for more than 3 seconds and stable for at least 10 seconds) or micro-arousal (increase in low-amplitude fast rhythms such as alpha oscillations or oscillations above 16Hz for less than 3 seconds) following the stimulus onset (see S8). Although micro-arousals were accompanied with behavioural responses in only a few cases, such trials were discarded from our analysis to ensure a conservative sleep scoring. This resulted in a total average of 70.8 trials per participant in this experiment, corresponding to 42.6 and 28.2 trials per participants in the wake and sleep conditions, respectively. Remaining trials were discarded (e.g. trials from the sleep list that were potentially associated with micro-arousals and/or with a button press). Among the trials included in the sleep condition, 79.4% were scored as NREM1 and 19.7% as NREM2. However, in order to satisfy standard definitions, NREM2 was scored only after the first occurrence of a *spontaneous* spindle or K-complex. As a consequence, evoked K-complexes or sleep spindles were still observed in 27.2% of NREM1, which reflects a deeper sleep stage than the standard NREM1. None of the participants reached the NREM3 stage or showed a REM episode. When considering a -2 to 4s

window around stimuli onset, K-complexes were observed in 24.5% of sleep trials (23.2% of NREM1 trials) and sleep spindles in 8.5% of sleep trials (4.9% of NREM1 trials).

Note that no consensus exists for a simple (e.g. scalar) criterion that can be used automatically to separate sleep from wake trials, arguably because of the individual differences in terms of amplitude/frequency range used to score vigilance states (see for instance [S7] for alpha and theta rhythms). For these reasons, the sleep assessment was performed by visual inspection, ensuring an evaluation that is both conservative (i.e., eliminating any sign of micro-arousal) and adaptative (i.e., taking into account individual variability). Nevertheless, to verify the validity of our sleep scoring methodology, we developed a scalar criterion that would constitute a quantitative evaluation of the difference between trials in the sleep and wake conditions. This scalar Vigilance Index (VI) was defined as the ratio of the mean power in specific frequency ranges computed on C3-C4 electrodes over each epoch (i.e., -2 to 6 seconds around stimulus onset), using a fast Fourier transform:

VI = [delta power + theta power + spindle power] / [alpha power + high-beta power]

With delta corresponding to 0.1 – 4 Hz, theta to 4 – 7 Hz, spindle frequency to 11 – 16 Hz, alpha to 8 – 13 Hz, and high-beta to 20 – 40 Hz). Low-Beta was not included as it overlaps with the frequency of spindles. For each epoch, power was normalized by the power in high frequency range (215 – 245 Hz). Delta, theta and spindle power being classically associated with sleep while alpha and high-beta are associated with wakefulness (see Figure S1 Panel B for an illustration), “sleep” trials should show higher VI values than “wake” trials. VI was computed for every trial in the sleep and wake conditions. The distribution of VI values across all trials was bi-modal ($p<0.01$, Hardigan Dip Test). Importantly, when considering VI values for “sleep” and “wake” trials separately, we checked that their respective distributions were statistically different ($p<0.001$, unpaired t-test). This was also true when considering subjects individually ($p<0.001$, unpaired t-test, Bonferroni correction). This demonstrates that we are genuinely dealing with two distinct brain states in our study.

Supplemental sleep scoring of Experiment 1 using standard guidelines. To ensure that our results did not reflect an underestimation of the level of sleepiness due to the sleep scoring method we used, and thus the possibility of missing potential contaminations by micro-arousals, we performed a re-scoring of our data using standard guidelines of the AASM [8]. Data were first continuously scored as wake, NREM1 and NREM2 using 20s epochs. Regular correct responses to stimuli, presence of alpha rhythms on occipital regions, eye-blinks or saccades were indicative of wakefulness. NREM1 was defined by the replacement of alpha rhythms with theta rhythms. Presence of slow eye movements, vertex sharp waves, evoked K-complexes or sleep spindles were also indicative of NREM1 onset. Finally, epochs showing spontaneous K-complexes or spindles were scored as NREM2. In order to retain as sleep trials only trials for which participants were and remained asleep, NREM1 and NREM2 trials associated with motor responses or micro-arousal (increase in low-amplitude fast rhythms lasting less than 3s) and arousals (e.g. associated or not with button presses) were discarded from further analysis. The corresponding LRP results are presented in figure S3.

Sleep assessment for Experiment 2. For Experiment 2, in which we directly compared a state of full alertness with NREM2, we relied exclusively on the standard sleep scoring method of the ASSM relying on 20-30 seconds epochs [S8] which is more adapted to the evaluation of deeper sleep states. In order to focus on NREM2, participants were assumed to be fully asleep if they were unresponsive and after the occurrence of the first spontaneous K-complex or sleep spindle (i.e. not appearing within at least 1 second follwing stimulus onset). There were also trials scored as NREM3 but those were not included in the final analysis as it concerned fewer trials and only a restricted set of participants (N=11). There was a total average of 147.7 trials per participant, corresponding to 68.7 and 79 trials in the wake and sleep conditions, respectively. The same procedure as for Experiment 1 was used here to discard trials micro-arousals and button presses.

Old/new recognition post-test. Experiment 2 was followed, after awakening, by an explicit recognition test in which they were presented, in random order, with spoken words that were

previously presented during the wake or sleep period, or new words that were not presented before. They were instructed to report, using one of two keys on a keyboard, whether the word was old (i.e., presented in the wake or sleep list) or new, without time pressure. Following their answer, they indicated their level of confidence on a scale ranging from 1 (completely guessing) to 7 (completely sure), again without time pressure. Each participant was presented with 108 words (36 words per condition). The old items from the sleep conditions that were subsequently scored as reflecting N1, (micro)-awakenings and/or button presses were discarded from the analysis, to match items used in the LRP analysis.

Supplemental references

S1. New, B., Pallier, C., Brysbaert, M., and Ferrand, L. (2004). Lexique 2: a new French lexical database. Behavior research methods, instruments, & computers : a journal of the Psychonomic Society, Inc *36*, 516-524.

S2. Brainard, D.H. (1997). The Psychophysics Toolbox. Spat Vis *10*, 433-436.

S3. Coles, M.G. (1989). Modern mind-brain reading: psychophysiology, physiology, and cognition. Psychophysiology *26*, 251-269.

S4. Maris, E., and Oostenveld, R. (2007). Nonparametric statistical testing of EEG- and MEG-data. Journal of neuroscience methods *164*, 177-190.

S5. Hori, T., Hayashi, M., and Morikawa, T. (1994). Topographical EEG changes and the hypnagogic experience. . Sleep Onset: Normal and Abnormal Processes, 237– 253.

S6. Tanaka, H., Hayashi, M., and Hori, T. (1997). Topographical characteristics and principal component structure of the hypnagogic EEG. Sleep *20*, 523-534.

S7. Klimesch, W., Doppelmayr, M., Schwaiger, J., Auinger, P., and Winkler, T. (1999). 'Paradoxical' alpha synchronization in a memory task. Brain Res Cogn Brain Res *7*, 493-501.

S8. Iber, C., Ancoli-Israel, S., Chesson, A., and Quan, S.F. (2007). The AASM Manual for the Scoring of Sleep and Associated Events: Rules, Terminology and Technical Specifications, (Westchester, IL: American Academy of Sleep Medicine).
